# Unmasking the burden and risk factors of leptospirosis in low- and middle-income countries: a systematic review and meta-analysis

**DOI:** 10.1016/j.onehlt.2026.101632

**Published:** 2026-10-24

**Authors:** Rachita Pradhan, Shishirendu Ghosal, Jyoti Ghosal, Swagatika Pati, Sneha Singh, Harshita Rajesh Dhusiya, Ankita Padhi, Debaprasad Parai, Matrujyoti Pattnaik, Hari Ram Choudhary, Anoop Velayudhan, Srikanta Kanungo, Debdutta Bhattacharya, Sanghamitra Pati

**Affiliations:** a Dept. of Microbiology & One Health, ICMR-National Institute of Health Research, Bhubaneswar, India; b Indian Council of Medical Research, New Delhi, India; c Academy of Scientific and Innovative Research (AcSIR), India

**Keywords:** Leptospirosis, Epidemiology, Risk factors, LMICs, Seroprevalence, Incidence, One Health

## Abstract

**Background:**

Leptospirosis is a prevalent yet neglected zoonotic disease originating from *Leptospira* sp.*,* posing a significant public health challenge in low- and middle-income countries (LMICs). Despite its significant impact on health outcomes, *Leptospira* infection remains under-diagnosed and under-reported due to non-specific clinical presentation and limited standardised diagnostic tests and capacity. Therefore, this study aimed to systematically synthesise available global evidence on incidence, prevalence (through serological tests) and risk factors associated with transmission of leptospirosis in LMICs.

**Methods:**

The review was prospectively registered on PROSPERO (CRD42023471312). Articles from Medline, Embase, ProQuest, CINAHL (Ebsco) and Google Scholar were systematically searched using MeSH/Emtree terms or CINAHL Headings, as appropriate, along with relevant keywords, till 23rd April 2024. Study quality was assessed using the Joanna Briggs Institute tool. Pooled estimates of incidence and seroprevalence were estimated using random-effects model, whenever appropriate.

**Results:**

After removing duplicates, 1391 of the 1955 articles were screened, with 46 articles meeting the inclusion criteria. Using the random effects model, the pooled incidence rate of leptospirosis was 56.86 per 1000 population per year and seroprevalence was reported to be 24.36% (highest in community-based studies: 29.53%). Key risk factors included jobs that involve close contact with livestock, poor housing and sanitation, and frequent exposure to rodents. Cases tend to spike during the monsoon season, when flooding and water contamination create ideal conditions for transmission.

**Conclusion:**

Leptospirosis remains an under-recognised public health concern in LMICs, characterised by widespread exposure and context-specific transmission dynamics. Strengthening surveillance, improving diagnostic capacity, and implementing targeted interventions are essential for effective disease control.

## Highlights

• This review synthesises leptospirosis burden and risk factors in LMICs.
• Community-based studies had the highest pooled seroprevalence (29.53%).
• Pooled incidence was 56.86 cases per 1000 population per year.
• Occupational and environmental exposures were associated with infection.
• Rainfall and flooding were associated with increased leptospirosis risk.

## 1 Introduction

Leptospirosis, caused by pathogenic spirochetes of the *Leptospira* genus, is a neglected tropical disease posing a significant public health risk, particularly in low- and middle-income countries (LMICs), where environmental and socioeconomic conditions facilitate transmission [1], [2]. *Leptospira* infection is widespread globally but remains under-recognised. The World Health Organisation (WHO) estimated 1.03 million global infections, 58,900 annual deaths, and 2.9 million disability-adjusted life years (DALYs) [3], [4]. However, these estimates are largely derived from modelling approaches [5] based on sparse, heterogeneous, and often facility-based LMIC data, with limited representation of community transmission and high-risk populations. Variations in diagnostic practices, case definitions, and surveillance capacity further constrain comparability and reliability. LMICs are particularly vulnerable to outbreaks because of converging socio-environmental and economic risk factors [6], [7], [8]. Leptospirosis is endemic in tropical and subtropical LMICs, including India, Brazil, and Indonesia, with burden highest during rains, when floods and standing water favour *Leptospira* survival and transmission *Leptospira* [9], [10], [11], [12]. Agricultural, animal-handling, sewage, and slaughterhouse workers are among those most at risk [11], [13], [14], [15]. Transmission occurs through direct contact with urine or tissues of infected animals or indirect exposure to contaminated soil or water [16], [17]. Rodents are principal reservoirs, excreting *LLeptospira* in urine and contributing significantly to environmental contamination, particularly in urban slums with sub-optimal sanitation and hygiene [2], [6], [7]. Livestock, including cattle and pigs, are important carriers in rural agricultural communities [2], [18]. Leptospirosis ranges from mild febrile illness to life-threatening complications like Weil's syndrome and severe pulmonary haemorrhagic syndrome - leptospirosis can present with a wide range of clinical symptoms. Its nonspecific clinical presentation, frequently leads to misdiagnosis as dengue, malaria, influenza and fever of unknown origin (FUO) [19]. Global burden analyses do not systematically synthesise context-specific risk factors or disaggregate epidemiological patterns across diverse LMIC settings, limiting their utility for targeted public health planning.

Such integrated strategies strengthen surveillance, enhance diagnostic capacity, and improve outbreak response, and are increasingly promoted as zoonotic disease management [20], [21], [22]. Despite its widespread presence and outbreak potential, leptospirosis remains largely underdiagnosed and underreported in LMICs due to non-specific clinical features, limited healthcare-provider awareness, and restricted access to confirmatory testing such as the Microscopic Agglutination Test (MAT) or polymerase chain reaction (PCR) [12]. Consequently, its actual incidence and prevalence remain poorly understood [23], [24]. Robust epidemiological data are critical for developing effective intervention strategies and to inform national health policies [11], [12]. Therefore, a systematic synthesis integrating disease burden and context-specific risk factors across LMICs is needed. Existing studies are often geographically restricted, heterogeneous, population-specific, limiting cross-setting comparability and generalisability. Furthermore, prior global estimates have not adequately captured the interplay among environmental, occupational, and socioeconomic determinants of transmission. Thus, public health planning and resource allocation remain inadequate, with lost opportunities for early intervention.

Hence, this review aims to systematically synthesise available evidence to generate consolidated estimates of leptospirosis incidence and seroprevalence across LMICs, and identify and categorise key environmental, occupational, and socioeconomic risk factors. Unlike prior modelling-based estimates [3], [4], this study provides disaggregated LMIC evidence, integrates burden with risk factor analysis, and incorporates community-level data to better characterise transmission dynamics.

## 2 Methodology

The protocol was prospectively registered with PROSPERO (CRD42023471312). The Preferred Reporting Items in Systematic Reviews and Meta-analysis (PRISMA, 2020) (Supplementary file 1) guideline was followed [25].

### 2.1 Search strategy

The search strategy includes five distinct concepts: a. Leptospirosis, b. Incidence, c. Prevalence, d. Risk factors, and (5) LMICs as MeSH terms. The initial strategy was developed for MEDLINE including the conventional MeSH and Emtree terms and CINAHL Headings along with more relevant keywords/entry terms from relevant articles - combined with appropriate Boolean operators (AND, OR). This search strategy was later modified for EMBASE and CINAHL (Supplementary File 2). The first ten pages of Google Scholar search results, comprising 100 records, were screened to identify additional relevant literature. *Leptospira*The databases were searched and downloaded for primary screening on 23rd April 2024.

#### 2.1.1 Inclusion criteria

Eligible studies included those that reported data on individuals diagnosed with leptospirosis or examined the incidence, seroprevalence, or risk factors of the disease at the population level. Only studies conducted in LMICs [World Bank classification, 2024] [26], were included. Studies were eligible if they provided observational data on leptospirosis incidence, seroprevalence, or risk factors. Only studies published in English were included.

#### 2.1.2 Exclusion criteria

Studies were excluded if they:

• reported incidence or seroprevalence data exclusively on animals;
• did not specify the population at risk or failed to provide the time period necessary for calculating incidence or prevalence; or
• were narrative reviews, scoping reviews, conference abstracts, or opinion articles.

No additional filters based on study design, publication year were applied beyond the eligibility criteria described.

### 2.2 Study selection

Articles retrieved from different databases were uploaded to the online software Rayyan [27] and the deduplications were sorted. The unique articles were screened in two stages by two independent researchers (RP and SS). The initial step involved screening titles and abstracts for relevance before conducting the full text screening with predetermined inclusion and exclusion criteria. Reviewer disagreements were settled through discussion with third coauthor (SG).

### 2.3 Data extraction

Relevant information on study characteristics (the number of new and old cases, the population at risk, time, and risk variables) was gathered using a standardised data extraction form (Supplementary file 3). Data was separately extracted by two reviewers (RP and SS), who then cross-checked the results to ensure consistency. Any disagreement was adjudicated by SG.

### 2.4 Risk of bias (RoB) assessment

The JBI Quality Assessment Tool for Observational Cohort and Seroprevalence Studies was used to evaluate the methodological quality and bias risk [28]. Each study was evaluated independently by two researchers (RP and SS) across domains, including sample size justification, outcome ascertainment, exposure measurement, and control for confounding. Studies were categorized as having low, moderate, or high risk of bias. Under the guidance of SG, disagreements regarding the inclusion or exclusion of articles at these stages were resolved through discussion.

### 2.5 Outcome

The primary outcomes of interest were incidence rate (cases per 1000 population per year), seroprevalence of leptospirosis in human populations, and risk factors associated with disease transmission. The definitions of Leptospirosis cases have been jotted, study-wise, in the data extraction sheet (Supplementary file 3). The measures of effect size were presented with 95% confidence intervals (CIs).

### 2.6 Data analyses

All statistical analyses were conducted using Stata version 18.0 (StataCorp, College Station, TX). Extracted data (number of individuals at risk, duration of follow-up, and new and old leptospirosis cases during the study period and population size) were used to calculate incidence rate and seroprevalence using the following formulae:

$$\frac{\mathrm{Number of}\;\mathrm{new}\;\mathrm{cases}}{\mathrm{Population size}\;\mathrm{at}\;\mathrm{risk}*\mathrm{Duration of the study}}\times 1000$$

$$\text{Seroprevalence}=\frac{\mathrm{No}.\mathrm{of cases}}{\mathrm{total population}}\times 100.$$

Seroprevalence analyses were stratified a priori by study setting (community-based, facility-based, and high-risk populations) and, where appropriate, by diagnostic method (e.g., MAT and ELISA).

*Meta Analyses*: Between-study heterogeneity was assessed using the Cochran Q statistic and quantified using the I^2^ statistic and between-study variance (τ^2^). Prediction intervals (PI) were calculated for pooled estimates to reflect the expected range of effects in individual future studies. Sensitivity analyses were conducted using leave-one-out methods to evaluate the robustness of pooled estimates. Subgroup analyses were performed where deemed appropriate.

*Meta Regression:* Exploratory univariable meta-regression analyses were conducted for seroprevalence outcomes when sufficient studies were available to examine the association between selected study-level characteristics (study setting, diagnostic method, country, and year of publication) and effect size. Meta-regression was not performed for analyses with limited numbers of studies (<10 studies) [29]. The detailed methodology for the data analyses has been described in Supplementary file 4.

### 2.7 Certainty of evidence assessment

The certainty of evidence for the pooled incidence and seroprevalence estimates was evaluated using an adapted Grading of Recommendations Assessment, Development and Evaluation (GRADE) framework. Five domains were considered: risk of bias, inconsistency, indirectness, imprecision, and publication bias. *Risk of bias* judgments were informed by the Joanna Briggs Institute (JBI) critical appraisal assessments, while *inconsistency* was evaluated using the magnitude and persistence of between-study heterogeneity, subgroup analyses, and sensitivity analyses. *Indirectness* was assessed in relation to the populations, settings, and geographical coverage of the included studies. *Imprecision* was considered in relation to the confidence intervals around the pooled estimates. *Publication bias* was explored using funnel-plot inspection and Egger's regression test. The overall certainty of evidence was intended to be categorized as high, moderate, low, or very low following explicit justification of the initial certainty level and any downgrading decisions.

### 2.8 Risk factor reporting

Studies reporting risk factors of leptospirosis transmission were synthesized using a narrative approach. A thematic content analysis was conducted to systematically identify, categorise and summarise emerging patterns. The seven thematic domains emerged are: (1) behavioural practices and personal hygiene, (2) occupational and recreational exposure, (3) household and environmental conditions, (4) climate and seasonality, (5) animal contact, (6) socio-economic determinants, and (7) surveillance mechanisms and intersectoral collaboration. Studies reporting incidence or seroprevalence were included for meta-analysis when at least three studies offered comparable outcome measures. For each study, data on exposures, effect sizes (odds ratios, relative risks) with 95% confidence intervals, study population, setting, and potential confounders were extracted and compiled into a structured risk factor evidence table (Supplementary file 5: supplementary tables 5 & 6).

## 3 Results

The initial search identified 1955 studies. After removing duplicates, 1391 unique studies were shortlisted for title and abstract screening. Among them, 85 were selected for full-text screening. Another 37 entries were excluded after failing to meet the purpose of this review, leading to inclusion of 46 unique studies (PRISMA flow diagram: Fig. 1).

**Fig. 1:**
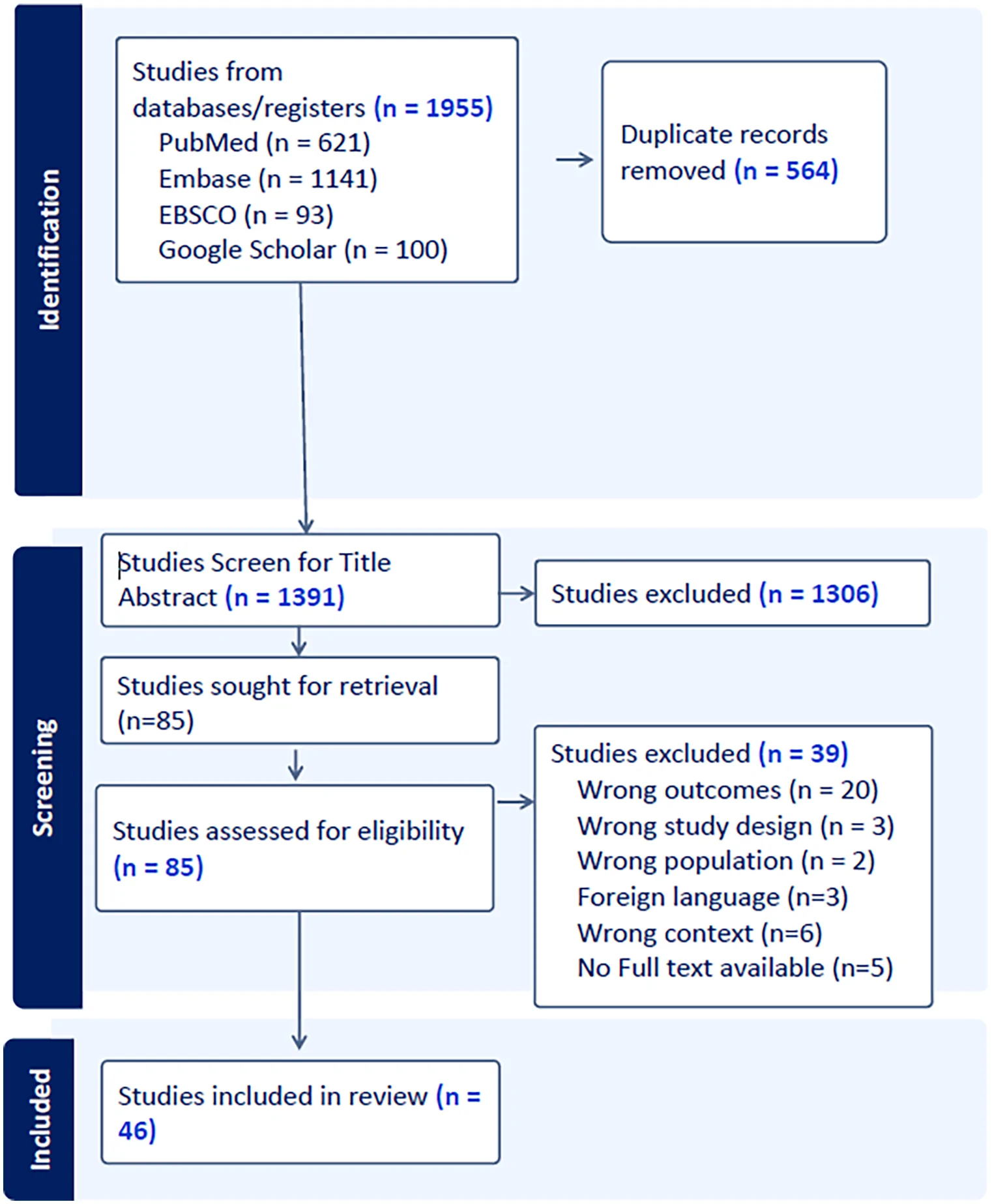
PRISMA flow diagram.

The included observational studies comprised of cross-sectional (*n* = 24), case-control (*n* = 19), and cohort designs (*n* = 5), conducted across India, Nepal, Bhutan, Brazil, Uganda, Egypt, Iran, Vietnam, Pakistan, Jordan, Rwanda, Tanzania, Lao PDR, and Sri Lanka. Sample sizes ranged from 42 to 1437 participants, with data collected between 2003 and 2021.The geographical distribution of study locations contributing to the incidence and seroprevalence meta-analyses is presented in Fig. 2.

**Fig. 2:**
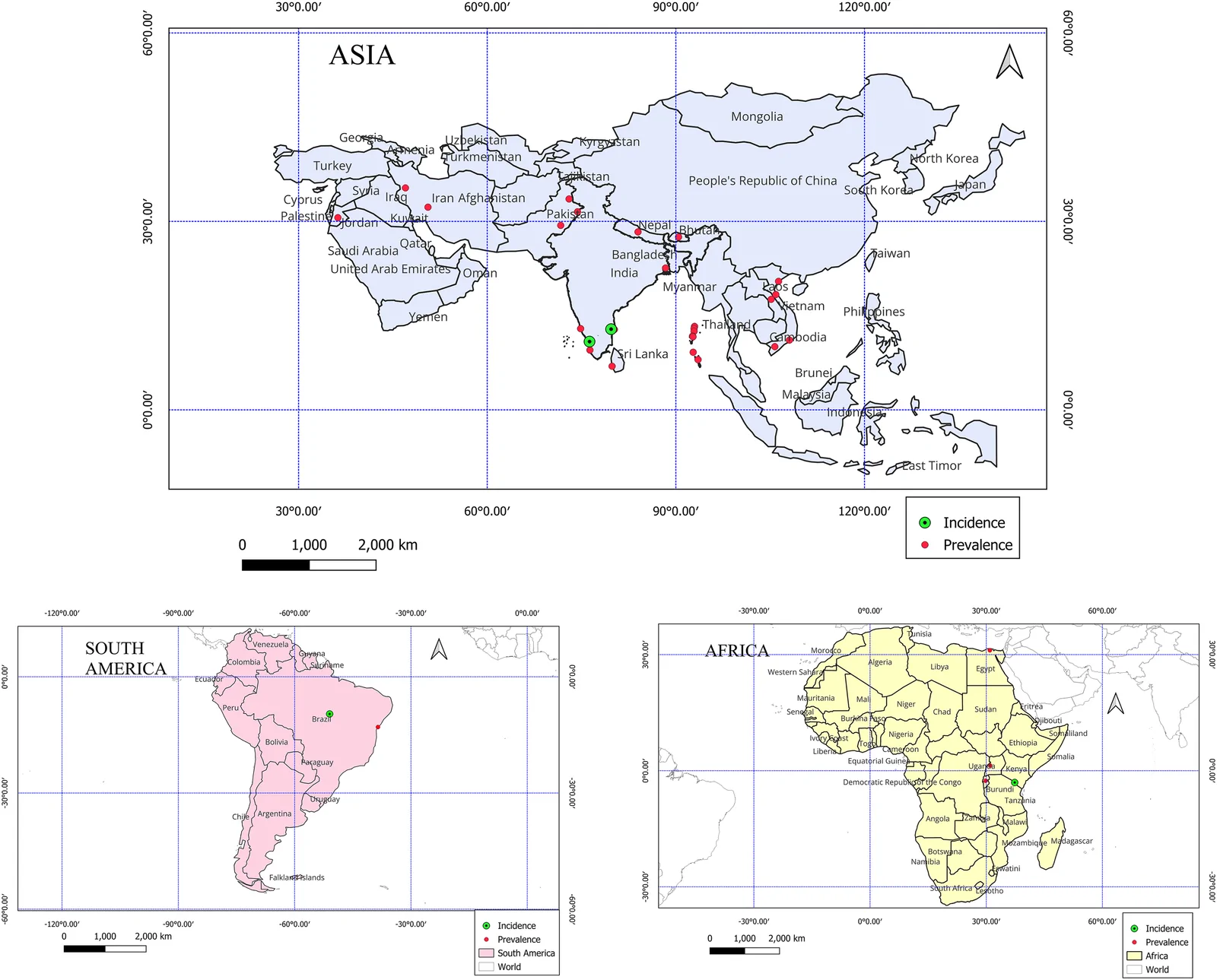
Geographical distribution of study locations contributing to the leptospirosis incidence and seroprevalence meta-analyses.

Most of the studies included for pooled prevalence were conducted both in urban & rural setting (*n* = 8), while seven and five studies concentrated only on urban and rural settings, respectively. Half of the prevalence studies ended before 2015, while the rest 10 commenced in of after 2015.

**Risk of Bias:**

Cohort studies included in the incidence meta-analysis demonstrated overall moderate risk of bias, with variability across domains. Key limitations were observed in participant selection, exposure measurement, confounding control, and follow-up completeness, which may influence the validity and representativeness of the findings (Fig. 3a).

**Fig. 3:**
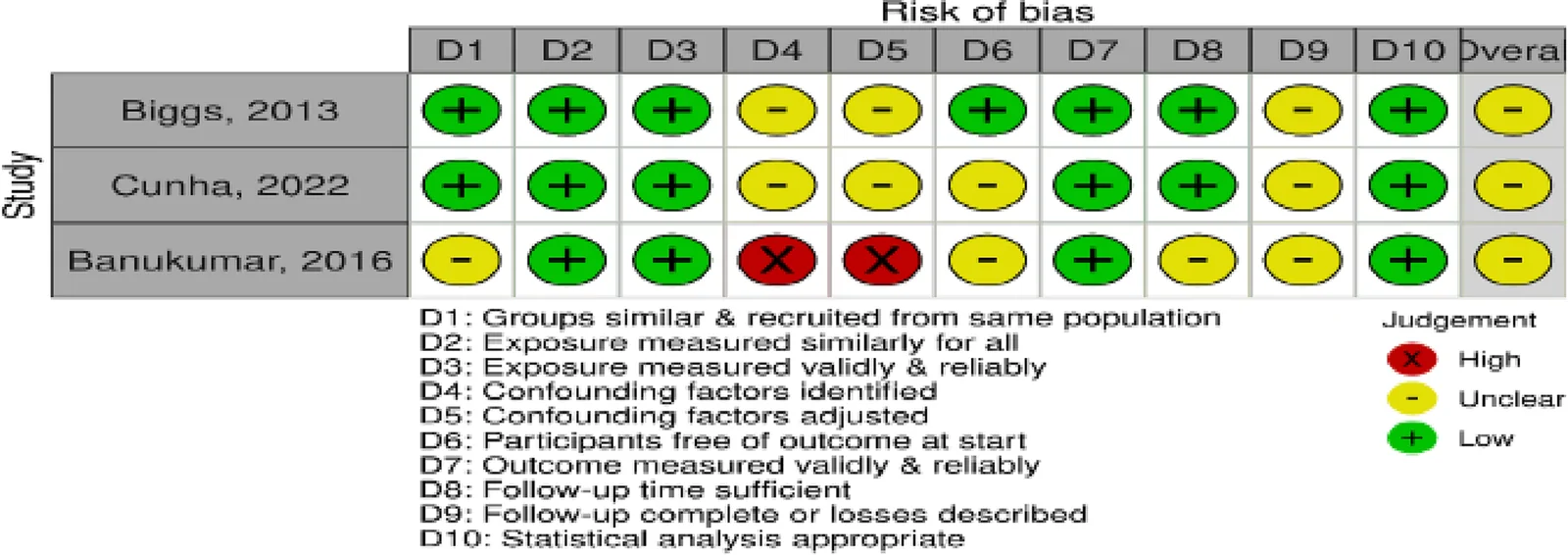

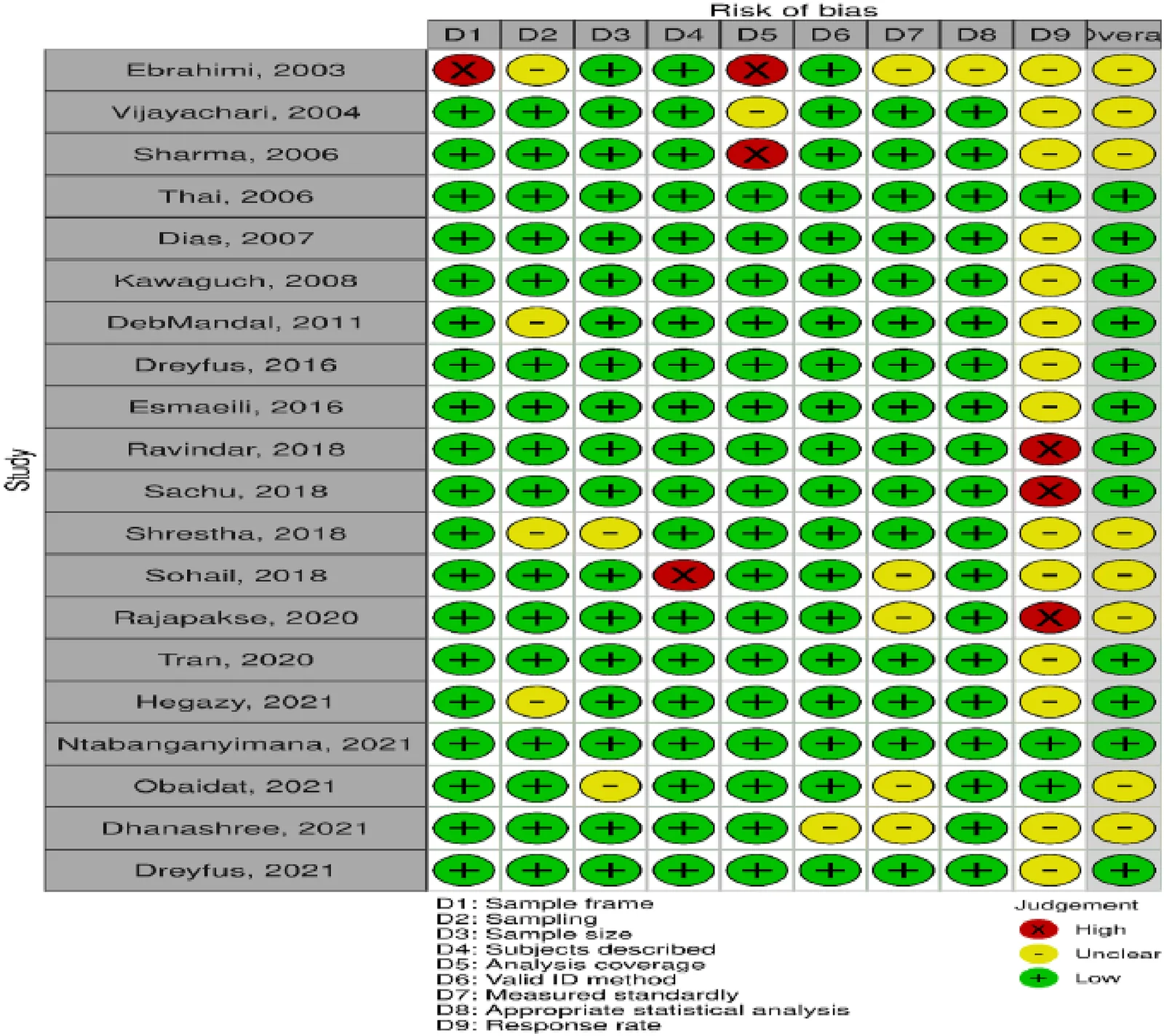
Risk of bias analysis.

Seroprevalence studies included in the meta-analysis demonstrated moderate to high methodological quality, with most domains rated as low risk of bias. Notable limitations were identified in analysis coverage and response rates, along with variability in sample size adequacy and measurement standardisation, which may affect the completeness and representativeness of the estimates (Fig. 3b).

### 3.1 Pooled incidence

The individual studies have reported a wide range of incidence rates, starting from 19.29 (95% CI:17.39, 21.18) to 471.79 (95% CI: 399.63, 543.95) per 1000 population per year [30], [31], [32], [33], [34]. Following the sensitivity analysis (described in detail in the Supplementary file 4), the final random-effects meta-analysis based on three cohort studies (from Brazil, India and Tanzania) yielded a pooled incidence rate of 56.86 cases per 1000 population per year (95% CI: 42.10, 71.62) (95% PI: −120.33, 234.05) (Fig. 4), with heterogeneity (I^2^) of 83.12% (*p* = 0.01) [30], [33], [34].

**Fig. 4:**
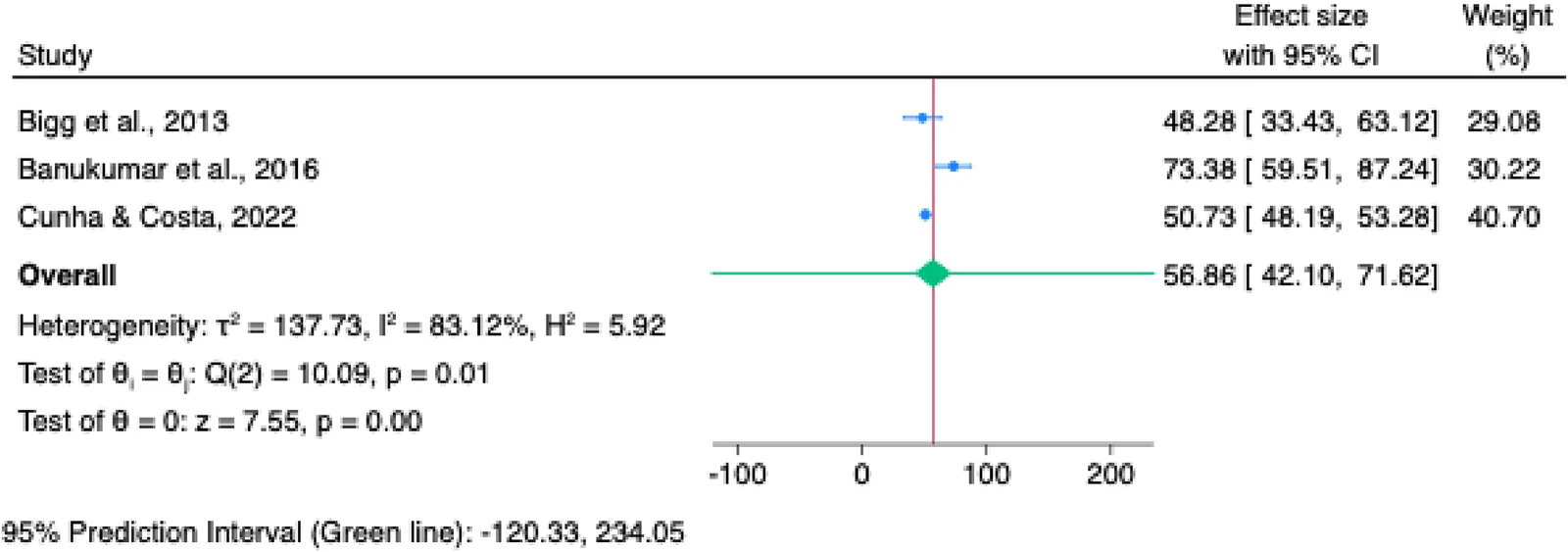
Pooled incidence rate (per 1000 per year).

### 3.2 Pooled seroprevalence

Twenty cross-sectional studies from diverse geographic regions (India, Nepal, Bhutan, Brazil, Uganda, Egypt, Vietnam, Iran, Pakistan, Jordan, Rwanda, Lao PDR, and Sri Lanka) were included to estimate the pooled seroprevalence of leptospirosis in LMICs [23], [35], [36], [37], [38], [39], [40], [41], [42], [43], [44], [45], [46], [47], [48], [49], [50], [51], [52], [53]. Study populations ranged from high-risk occupational groups (farmers, cattle handlers, butchers/slaughterhouse workers) [23], [39], [51] to school-going children, healthy community residing in endemic areas [52], [53], and patients presenting with acute febrile illness at tertiary hospitals [40,45]The random-effects model yielded a pooled seroprevalence of 24.36% (95% CI: 16.64%, 32.08%) (95% PI: −13.34, 62.06) with 99.52% heterogeneity (I^2^) (*p* < 0.001) (Fig. 5).

**Fig. 5:**
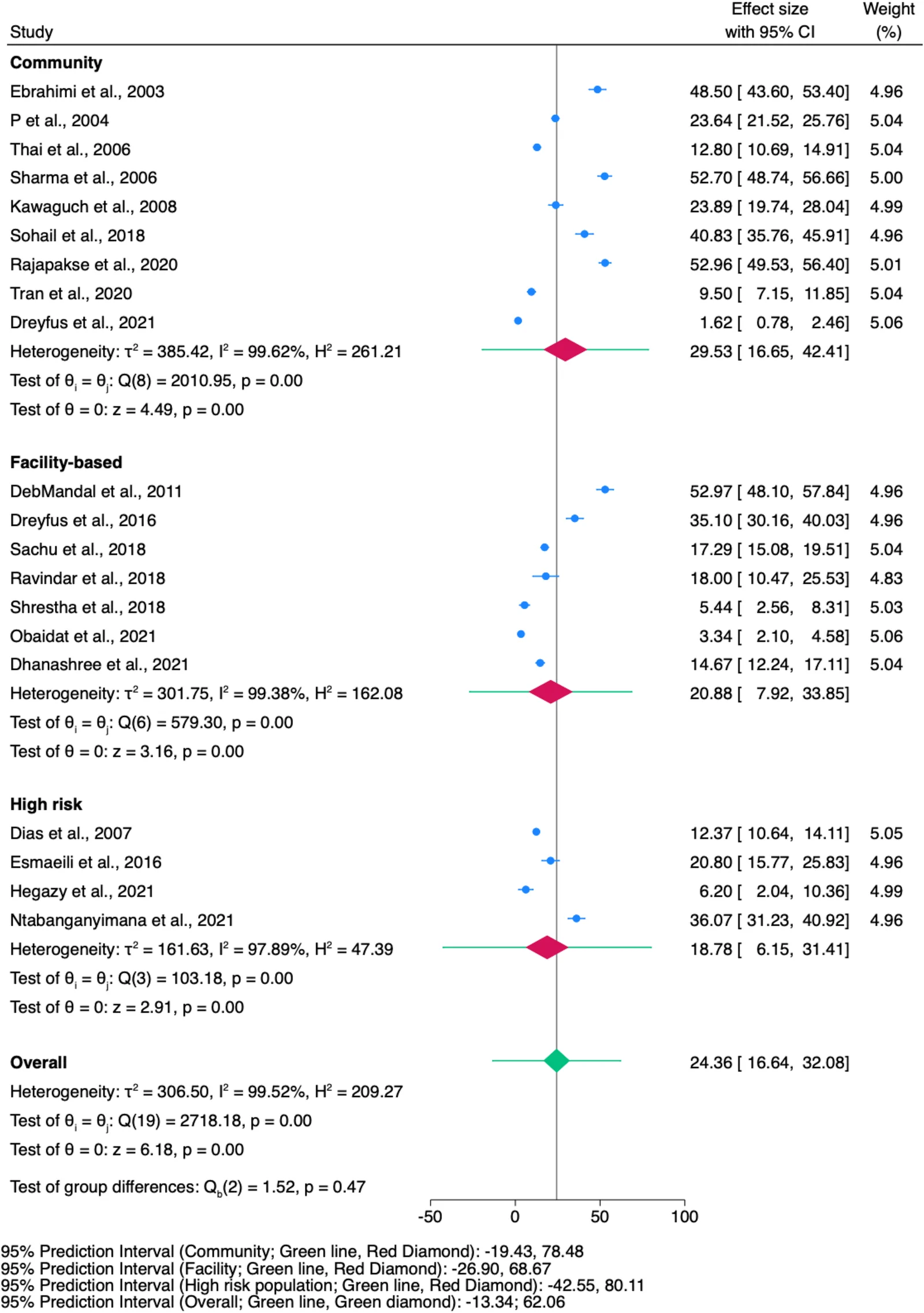
Pooled seroprevalence among the population subgroups [Community (*n* = 9), Facility (*n* = 7) and High-risk population (*n* = 4)].

Given the high heterogeneity, the seroprevalence was stratified by three study settings: (i) Community-based, (ii) Facility-based and (iii) based on high-risk populations. Nine community-based studies yielded the pooled seroprevalence of 29.53% (95% CI: 16.65, 42.41) (95% PI: −19.43, 78.48), with 99.62% heterogeneity (*p* < 0.001). Facility-based studies (*n* = 7), showed an overall seroprevalence of 20.88% (95% CI: 7.92, 33.85) (95% PI: −26.90, 68.67) (I^2^ = 99.38%, *p* < 0.001). Meta-analysis of four studies on high-risk occupational groups reported a pooled seroprevalence of 18.78% (95% CI: 6.15, 31.41) (95% PI: −42.55, 80.11), with high heterogeneity (I^2^ = 97.89%, *p* < 0.001) (Fig. 5). Sensitivity analysis using the leave-one-out method was carried out for all the respective groups, where the between-study heterogeneity as well as the pooled estimates remained unchanged. Following this, a subgroup analysis was conducted based on diagnostic methods (ELISA vs. MAT) for the community and facility-based studies (due to inadequate sample size, subgroup analyses was not performed in the high-risk group). Upon stratification, heterogeneity also remained high. The detailed description has been illustrated in the Supplementary file 4.

### 3.3 Publication bias

The funnel plot (Fig. 6) shows some visual asymmetry, with studies distributed somewhat unevenly around the pooled effect estimate. However, this visual asymmetry is not supported by Egger's test (z = −0.11, *p* = 0.911), indicating no statistically significant evidence of small-study effects. Therefore, based on the funnel plot and Egger's test together, there is no strong evidence of publication bias among the prevalence studies.

**Fig. 6:**
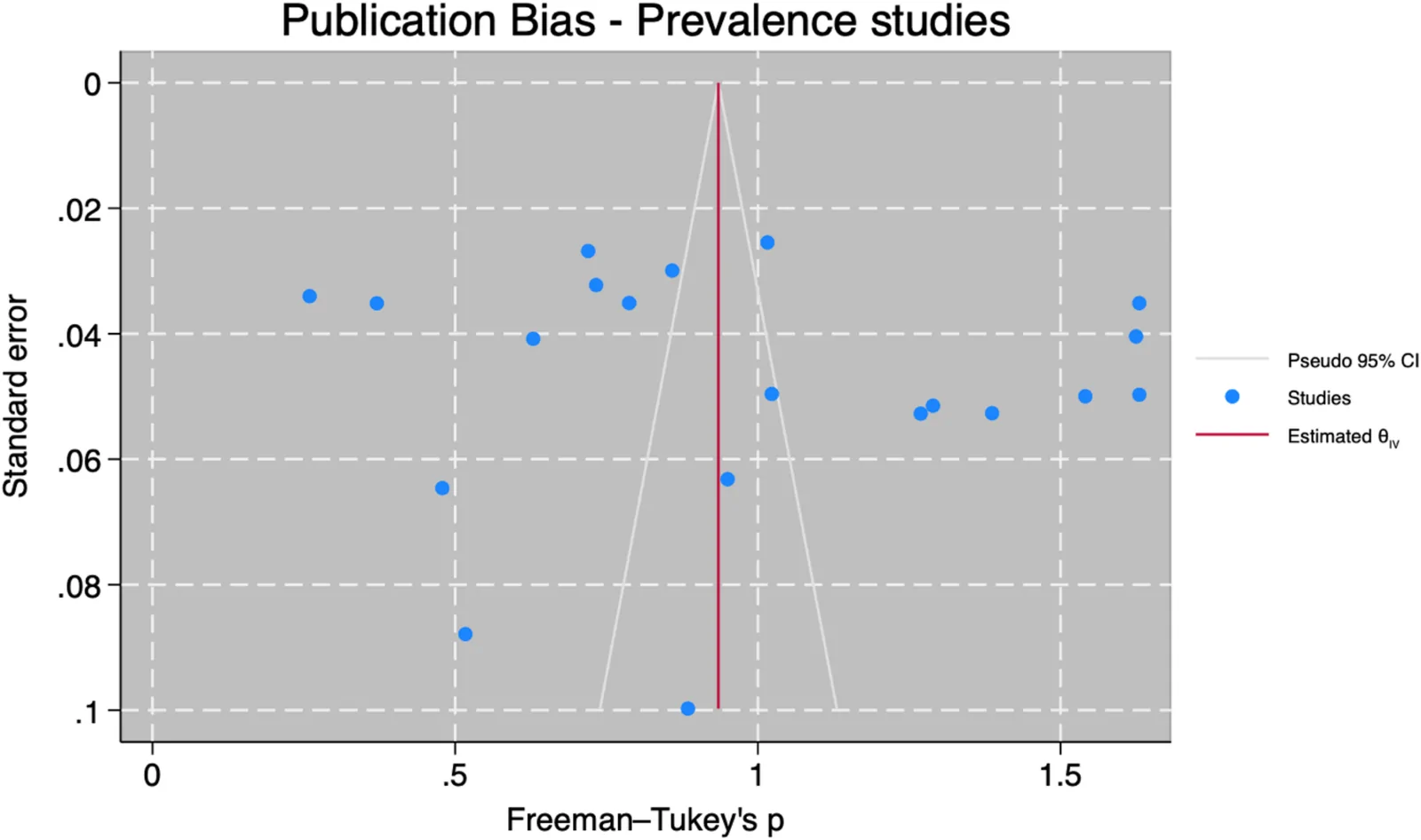
Publication bias for the Prevalence studies **(*n*** **=** **20).**

### 3.4 Certainty of evidence assessment

The certainty of evidence for incidence and seroprevalence of leptospirosis was assessed using an adapted GRADE framework (Supplementary table 3 & 4).

#### 3.4.1 Certainty of evidence assessment for incidence of leptospirosis in LMICs

Three cohort studies contributed to the final meta-analysis, yielding a pooled incidence rate of 56.86 cases per 1000 population per year (95% CI: 42.10–71.62), with substantial between-study heterogeneity (I^2^ = 83.12%). The overall certainty of evidence was rated as very low (vide supplementary table 3 (supplementary file 4) for detailed description) because of concerns regarding risk of bias, inconsistency, indirectness, and imprecision.

#### 3.4.2 Certainty of evidence assessment for seroprevalence of leptospirosis in LMICs

The overall pooled seroprevalence across 20 studies was 24.36% (95% CI: 16.64–32.08%), with substantial between-study heterogeneity (I^2^ = 99.52%). The certainty of evidence was rated as very low, primarily due to concerns regarding risk of bias, inconsistency, and imprecision. The detailed explanation has been illustrated in the supplementary table 4.

### 3.5 Risk factor analysis

The narrative synthesis identified behavioural, occupational, environmental, animal-related, socioeconomic, and climatic factors associated with risk to *Leptospira* infection, often context-specific yet consistent across settings. Findings were summarised (Supplementary file 5: supplementary table 5 & 6) in a structured manner and other relevant details (Study setting, Exposure, Population, Association of leptospirosis with that particular exposure and Direction of association) have been described in Table S4 domain-wise.

#### 3.5.1 Behavioural and personal hygiene

In Vietnam, drinking unboiled water increased risk to leptospirosis (OR: 1.27) [54] An Iranian study highlighted elevated risk from exposure to stagnant rice field water while face washing or through skin abrasions [55]. Among slaughterhouse workers in Kenya, wounds (OR: 3.1) and eating at work (OR: 2.1) were associated with increased odds of seropositivity [56]. Protective practices included handwashing after farming or gardening (OR: 0.57), and showering after livestock or pets contact [53], [54].

#### 3.5.2 Occupational and recreational exposure

Farming-related occupations were frequently associated with increased risk, including farmers (OR: 8.26), specifically, rice cultivators (OR: 2·83). Animal husbandry, particularly pig (OR: 10·48) or cat ownership(OR: 2·62), further increased susceptibility [54]. High seroprevalence was observed among military personnel, kennel workers, waste management employees, individuals exposed to sewage or wound workers [57], [58]. Slaughterhouse workers, butchers, veterinarians and forestry workers (RR: 9·2) were consistently identified as high-risk groups [39], [59], [60], [61]. Cleaning offal among slaughterhouse workers in Kenya was also associated with increased odds of seropositivity (OR: 5.1) [56].). In Sri Lanka, handling cattle (OR: 3.57) and having an animal farm near the home or workplace (OR: 3.86) were associated with increased odds of infection [62].

#### 3.5.3 Household and environmental conditions

Studies from India reported higher risk among households with mud floors, leaky roofs, and proximity to open sewers or flood-prone areas [62], [63], [64]. Slum settings with overcrowding, rodent infestations, poor sanitation, and waste accumulation further increased vulnerability [6], [65]. Environmental exposures, such as mud puddles (OR: 3.67), water ditches (OR: 3.0), and a history of flooding (OR: 2.68), were also associated with leptospirosis [66]. Contamination of rivers, rice fields, and wells in Boyolali (Indonesia) demonstrated key environmental reservoirs [67].

#### 3.5.4 Animal and rodent contact

Animal contact was a consistent predictor of *Leptospira* infection. Exposure to pigs (OR: 10.48), cattle (OR: 3.57), poultry (OR: 3.6), pet rats (RR: 13.9), and dogs substantially increased risk [40], [48], [49], [50], [51]. Livestock-related activities like milking, birthing assistance, slaughtering, and cleaning animal waste (urine) were also associated with infection (OR: 3.57) [38], [68].

#### 3.5.5 Socioeconomic factors

Low socioeconomic status, limited education, overcrowding, and suboptimal sanitation were major risk drivers [6], [37]. Seroprevalence was inversely associated with schooling and income [37]. In rapidly urbanising settings, poverty further amplified the risk [65]. [65]. In a disadvantaged community in Brazil, participants aged over 40 years (OR: 3.45) and employed participants (OR: 2.45) had higher odds of seropositivity [69]. However, piped water access and rat sightings were not uniformly associated (OR: 0.9) with seropositivity [37].

#### 3.5.6 Climate and seasonality

In Salvador, Brazil, Leptospirosis incidence peaked during the monsoon season (April–July), with rainfall exceeding normal by 20 mm increasing risk by 12% [33]. Additionally, high humidity were consistently linked to elevated seroprevalence [46]. Flooding, particularly among women exposed to floodwaters [51] and prolonged standing in water were important risk factors [59].

#### 3.5.7 Surveillance and intersectoral collaboration

One included study highlighted the relevance of the One Health approach, emphasising integrated surveillance across human, animal, and environmental sectors. Environmental surveillance in Dadra and Nagar Haveli in India demonstrated how synchronised monitoring improves outbreak detection and timely response. These findings underscore the need to institutionalise intersectoral collaboration within routine disease surveillance systems [70].

## 4 Discussion

This systematic review and meta-analysis synthesized evidence on the burden and risk factors of leptospirosis across LMICs. The estimated pooled incidence was 56.86 cases per 1000 populations per year (95% CI: 42.10–71.62), based on three cohort studies with considerable variation, while pooled seroprevalence (from 20 studies) was notably low (24.36%). Seroprevalence was higher in community-based studies compared to facility-based studies (29.53% vs 20.88%) - indicating substantial population-level exposure across different geographies. These differences in estimates may be influenced by between-study heterogeneity in study populations, diagnostic approaches, and uneven geographic representation of the available evidence [3], [6], [7], [8]. While estimating the pooled incidence, substantial heterogeneity (I^2^ = 83.12%) was observed; the prediction interval (PI) was wide as well (−120.33 to 234.05). The negative lower bound is a statistical consequence of calculating PI on an absolute scale and does not imply a biologically possible negative incidence. Hence, the pooled estimates required utmost caution while interpreting and referring for further investigation. In particular, the pooled incidence should not be interpreted as representative of the incidence across all LMICs.

The pooled seroprevalence of 24.36%, which is comparable to other endemic regions, including Malaysia and sub-Saharan Africa [71], [72], indicates a substantial evidence of previous or recent exposure to leptospira. However, this estimate was tagged along with a very high between-study heterogeneity (I^2^ = 99.52%). The stratified analysis revealed a higher seroprevalence in community-based studies compared to facility-based studies (29.53% vs 20.88%) - possibly due to including mild or asymptomatic infections from the community settings, which are often missed by hospital-based surveillance systems [3], [4], capturing primarily acute febrile and symptomatic cases, potentially underestimating the true population-level burden. Similar observations from previous studies emphasised that community-level surveillance is critical for accurate burden estimation, facilitate prevention effective case detection and timely outbreak detection in endemic LMICs [73], [74], [75]. These findings support the integration of community- and hospital-based data to capture the broader spectrum of leptospirosis infection and to better inform public health decision-making.

The substantial heterogeneity observed in pooled seroprevalence also needs to be considered in the context of the uneven geographic representation of available evidence, which may limit the generalizability of burden estimates. Heterogeneity in pooled seroprevalence was addressed through stratification by study setting, diagnostic methods, geographic location, and study period. Leave-one-out sensitivity analyses did not materially alter the pooled estimates or between-study heterogeneity, suggesting that the observed variability was not driven by a single influential study. The included studies used different serological approaches, principally MAT and ELISA, and differences in assay characteristics, interpretation criteria and case definitions may contribute to variation in observed seropositivity. Although stratification by diagnostic method was undertaken, substantial heterogeneity remained within the resulting groups. The observed heterogeneity underscores the importance of standardised diagnostic methods and harmonized case definitions to generate more comparable prevalence estimates across studies [16].

The review identified consistent risk factors across the seven distinct domains. Behaviours like consuming untreated water and exposure to stagnant water, occupational activities involving livestock or animal products, inadequate sanitation facilities, poor housing conditions and contact with rodent and their excreta were consistently associated with leptospirosis [6], [10], [37], [38]. Leptospirosis transmission peaks during the monsoon seasons, the timing of which varies across countries and regions. Flooding, high humidity and prolonged exposure to stagnant water, can increase contact with contaminated environments, particularly in areas with poor sanitation and high animal exposure [33], [51], [59], [76], especially where drainage and waste management are inadequate [9], [10]. These dynamics, combined with inadequate intersectoral collaborative supervision, underscore the importance of implementing integrated One Health strategies that address human, animal, and environmental factors simultaneously. The consistency of these risk factors reinforces their broader relevance while emphasising the potential value of coordinated surveillance across human, animal and environmental sectors [20], [21], [22], [70]. An integrated surveillance system could combine human case detection with veterinary surveillance, environmental monitoring and information on rainfall, flooding and other ecological conditions to support earlier identification of transmission hotspots. Such cross-sectoral approaches may be particularly valuable in LMICs where environmental and health-system vulnerabilities frequently overlap [75]. Although One Health approaches were limited in the included literature, the evidence across LMICs provides a valuable framework for strengthening future prevention, surveillance and intervention strategies [20], [70].

**Strength & Limitations:** Strength of this Systematic Review and Meta Analysis lies in inclusion of multiple databases, complemented by Goolgle Scholar search. The synthesis considered incidence, seroprevalence and risk factors together, allowing epidemiological burden to be interpreted alongside potential transmission determinants. The inclusion of sub-group analysis provides a broader perspective on the epidemiology of leptospirosis complemented by prediction intervals, leave-one-out sensitivity analyses and meta regression. The limitations include:

a. substantial heterogeneity across studies, limiting the precision, generalisability and comparability of pooled incidence rate and prevalence.
b. uneven geographic representation, with greater representation from India and Brazil.
c. predominantly hospital-based incidence studies, potentially missing mild or asymptomatic community infections; and
d. limited age- and sex-disaggregated data, restricting assessment of demographic differences.

**Policy implications and way forward**: The findings of this review have several important policy implications. First, surveillance systems in LMICs should be strengthened by expanding community-based surveillance and integrating human, animal and environmental health data [71], [73], [74]. Such integration would enable early outbreak detection and more accurate estimation of disease burden, particularly in underserved and high-risk regions.

Second, enhancing access to reliable and standardised diagnostic tools, such as the Microscopic Agglutination Test (MAT) and polymerase chain reaction (PCR), is essential to reduce misdiagnosis and underreporting [12], [16]. Investment in laboratory infrastructure and decentralised diagnostic capacity, especially in peripheral and rural health settings, should be prioritised.

Third, targeted environmental and occupational risk mitigation strategies are necessary. These should include improvements in water, sanitation, and hygiene (WASH), management of flooding and waste disposal, rodent control measures and protective measures for high-risk occupational groups such as agricultural and sanitation workers [6], [10], [11]. Seasonal preparedness strategies, particularly during the monsoon in flood-prone areas, should also be incorporated into public health planning [33], [76].

Finally, leptospirosis control requires sustained One Health coordination. Human surveillance should be linked with veterinary surveillance, environmental monitoring and relevant meteorological information to identify emerging transmission risks and support timely intervention [20], [21], [22], [70], [75].

Future research should priorities longitudinal cohort studies to improve incidence estimates and transmission dynamics. Studies in underrepresented LMIC regions, particularly in Africa and parts of Southeast Asia are also needed. Harmonized diagnostic criteria and reporting of assay type and positive thresholds would improve comparability across studies. Additionally, geospatial mapping of high-risk areas and evaluation of cost-effective, community-based interventions will be essential to guide resource allocation and policy decision-making.

## 5 Conclusion

This study systematically quantifies the incidence and seroprevalence of leptospirosis in LMICs, demonstrating that it represents a significant public health burden. This study provides indicative estimates of leptospirosis burden and highlights consistent risk factors, while underscoring critical gaps in surveillance and evidence across LMICs. The findings underscores the importance of strengthening public health surveillance system by improving early detection and diagnosis to enable prompt treatment and thereby halt transmission. Moreover, strengthening intersectoral collaboration through a coordinated One Health approach, alongside sustained investment in public health infrastructure, will be pivotal for preventing, and reducing the global burden of leptospirosis.

## CRediT authorship contribution statement

**Rachita Pradhan:** Writing – original draft, Software, Methodology, Formal analysis, Data curation. **Shishirendu Ghosal:** Writing – original draft, Visualization, Methodology, Formal analysis. **Jyoti Ghosal:** Writing – original draft, Visualization, Formal analysis. **Swagatika Pati:** Writing – original draft, Data curation. **Sneha Singh:** Writing – original draft, Methodology. **Harshita Rajesh Dhusiya:** Writing – original draft, Data curation. **Ankita Padhi:** Writing – original draft. **Debaprasad Parai:** Writing – review & editing, Validation, Methodology. **Matrujyoti Pattnaik:** Writing – review & editing, Visualization. **Hari Ram Choudhary:** Writing – review & editing. **Anoop Velayudhan:** Writing – review & editing. **Srikanta Kanungo:** Writing – review & editing, Supervision. **Debdutta Bhattacharya:** Writing – review & editing, Supervision, Methodology, Conceptualization. **Sanghamitra Pati:** Writing – review & editing, Supervision, Conceptualization.

## Ethical approval

Ethical approval was not required for this study as it is a systematic review and meta-analysis of previously published data, and no human subjects were directly involved.

## Declaration of competing interest

The authors declare that they have no known competing financial interests or personal relationships that could have appeared to influence the work reported in this paper.

## Data Availability

All data were derived from previously published studies cited in the manuscript. Extracted data and supporting materials are available within the article and its supplementary files.

## Appendix A Supplementary data

Supplementary material 1. PRISMA checklist

Supplementary material 2. Search strategy

Supplementary material 3. Data extraction sheet

Supplementary material 4. Subgroup analysis

Supplementary material 5. Leptospirosis risk-factor evidence table
