# Supplementary material for: Unmasking the burden and risk factors of leptospirosis in low- and middle-income countries: a systematic review and meta-analysis
Source: One Health. 2026 Oct 24;23:101632. doi: 10.1016/j.onehlt.2026.101632 (PMC13742084; doi:10.1016/j.onehlt.2026.101632)
Supplement: Supplementary file 2 [file mmc2.docx]

| ***Search Strategy for PubMed*** | | **Date of search:**  23.04.2024 |
| --- | --- | --- |
| **Concept** | **Search Terms** | **Result** |
| **Concept 1: Leptospirosis**  **#1** | **((((((((((((((((((((((((((((((((("Leptospirosis"[Mesh]) OR (Leptospiroses[Title/Abstract])) OR (Leptospira Infection[Title/Abstract])) OR (Infection, Leptospira[Title/Abstract])) OR (Infections, Leptospira[Title/Abstract])) OR (Leptospira Infections[Title/Abstract])) OR (Stuttgart Disease[Title/Abstract])) OR (Mud Fever[Title/Abstract])) OR (Fever, Mud[Title/Abstract])) OR (Rice-Field Fever[Title/Abstract])) OR (Fever, Rice-Field[Title/Abstract])) OR (("oryza"[MeSH Terms] OR "oryza"[All Fields] OR "rice"[All Fields]) AND "field fever"[Title/Abstract])) OR ("Cane-Cutter"[All Fields] AND "Fever"[Title/Abstract])) OR ((("canes"[MeSH Terms] OR "canes"[All Fields] OR "cane"[All Fields]) AND ("cutter"[All Fields] OR "cutters"[All Fields])) AND "Fever"[Title/Abstract])) OR (("fever"[MeSH Terms] OR "fever"[All Fields] OR "fevers"[All Fields]) AND "Cane-Cutter"[Title/Abstract])) OR (Swineherd's Disease[Title/Abstract])) OR (Swineherd's Diseases[Title/Abstract])) OR (Canicola Fever[Title/Abstract])) OR (Fever, Canicola[Title/Abstract])) OR (Leptospira Canicola Infection[Title/Abstract])) OR (Infections, Leptospira Canicola[Title/Abstract])) OR (Leptospira Canicola Infections[Title/Abstract])) OR (Leptospirosis Canicola[Title/Abstract])) OR (leptospirosis canicola[Title/Abstract])) OR ("Weil Disease"[Mesh])) OR (Disease, Weil[Title/Abstract])) OR (Leptospirosis, Icterohemorrhagic[Title/Abstract])) OR (Icterohemorrhagic Leptospirosis[Title/Abstract])) OR (Weil's Disease[Title/Abstract])) OR (Disease, Weil's[Title/Abstract])) OR (Weils Disease[Title/Abstract])) OR (Jaundice, Spirochetal[Title/Abstract])) OR (Spirochetal Jaundice[Title/Abstract]** | 9,730 |
| **Concept 2.1 : Incidence**  **#2** | ((((((((((((((((((((((((((Incidence[Mesh]) OR ("Rate, Person-time"[Title/Abstract])) OR ("Person-time Rates"[Title/Abstract])) OR (Person time Rate"[Title/Abstract])) OR ("Person-time Rate"[Title/Abstract])) OR ("Rate, Incidence"[Title/Abstract])) OR (OR "Incidence Rates"[Title/Abstract])) OR ("Incidence Rate"[Title/Abstract])) OR ("Incidence, Cumulative"[Title/Abstract])) OR ("Cumulative Incidences"[Title/Abstract])) OR ("Cumulative Incidence"[Title/Abstract])) OR ("Rate, Attack"[Title/Abstract])) OR ("Attack Rates"[Title/Abstract])) OR ("Attack Rate"[Title/Abstract])) OR ("Proportion, Incidence"[Title/Abstract])) OR ("Incidence Proportions"[Title/Abstract])) OR ("Incidence Proportion"[Title/Abstract])) OR ("Secondary Attack Rates"[Title/Abstract])) OR ("Rate, Secondary Attack"[Title/Abstract])) OR ("Rate, Secondary Attack"[Title/Abstract])) OR ("Attack Rate, Secondary"[Title/Abstract])) OR ("Secondary Attack Rate"[Title/Abstract])) OR (Incidences[Title/Abstract])) OR ("Incidence"[Title/Abstract])) OR (distribution*[Title/Abstract])) OR (Epidemiology[Title/Abstract])) OR ("incidence rate*"[Title/Abstract]) | 2,371,505 |
| **Concept 2.2: Prevalence**  **#3** | **(((((((("Prevalence"[MeSH]) OR ("Prevalences"[Title/Abstract])) OR ("Period prevalence"[Title/Abstract])) OR ("Period prevalences"[Title/Abstract])) OR ("prevalence period"[Title/Abstract])) OR ("Point Prevalence"[Title/Abstract])) OR ("Point Prevalences"[Title/Abstract])) OR ("prevalence point"[Title/Abstract])) OR ("Sero-Prevalence"[Title/Abstract])** | 3,65,276 |
| **Concept 2.3: Risk factors**  **#4** | ((((((((((((((((((((((((((((((((((("Risk Factors"[Mesh]) OR ("Factor, Risk"[Title/Abstract])) OR ("Risk Factor"[Title/Abstract])) OR ("Social Risk Factors"[Title/Abstract])) OR ("Factor, Social Risk"[Title/Abstract])) OR ("Factor, Social Risk"[Title/Abstract])) OR ("Factors, Social Risk"[Title/Abstract])) OR ("Risk Factor, Social"[Title/Abstract])) OR ("Risk Factors, Social"[Title/Abstract])) OR ("risk factors*"[Title/Abstract])) OR ("Social Risk Factor"[Title/Abstract])) OR ("Health Correlates"[Title/Abstract])) OR ("Correlates, Health"[Title/Abstract])) OR ("Population at Risk"[Title/Abstract])) OR ("Populations at Risk"[Title/Abstract])) OR ("Risk Scores"[Title/Abstract])) OR ("Risk Score"[Title/Abstract])) OR ("Score, Risk"[Title/Abstract])) OR ("Risk Factor Scores"[Title/Abstract])) OR ("Risk Factor Scores"[Title/Abstract])) OR ("Risk Factor Score"[Title/Abstract])) OR ("Score, Risk Factor"[Title/Abstract])) OR (Exposure*[Title/Abstract])) OR ("Health risk"[Title/Abstract])) OR (Vulnerability*[Title/Abstract])) OR (Threat*[Title/Abstract])) OR ("risk factor*"[Title/Abstract])) OR ("Risk Quotient"[Title/Abstract])) OR (Determinant*[Title/Abstract])) OR (Predictor*[Title/Abstract])) OR (Cause*[Title/Abstract])) OR (Contributor*[Title/Abstract])) OR (Influence*[Title/Abstract])) OR ("Associated factor*" [Title/Abstract])) OR (Covariates*[Title/Abstract])) OR (Correlates*[Title/Abstract]) | 7,426,228 |
| **Concept 3: LMICs**  **#5** | **(((((((((((((((((((((((((((((((((((((((((((((((((((((((((((((((((((((((((((((((((((Afghanistan[tiab]) OR ("Burkina Faso"[tiab])) OR (Burundi[tiab])) OR ("Central African Republic"[tiab])) OR (Chad[tiab])) OR (Eritrea[tiab])) OR (Ethiopia[tiab])) OR (Gambia[tiab])) OR (Guinea[tiab])) OR ("Guinea-Bissau"[tiab])) OR ("Democratic People's Republic of Korea"[tiab])) OR (Liberia[tiab])) OR (Madagascar[tiab])) OR (Malawi[tiab])) OR (Mali[tiab])) OR (Mozambique[tiab])) OR (Niger[tiab])) OR (Rwanda[tiab])) OR ("Sierra Leone"[tiab])) OR (Somalia[tiab])) OR ("South Sudan"[tiab])) OR (Sudan[tiab])) OR (Syria[tiab])) OR (Togo[tiab])) OR (Uganda[tiab])) OR (Yemen[tiab])) OR (Angola[tiab])) OR (Honduras[tiab])) OR (Philippines[tiab])) OR (Algeria[tiab])) OR (India[tiab])) OR (Samoa[tiab])) OR (Bangladesh[tiab])) OR (Indonesia[tiab])) OR ("Sao Tome and Principe"[tiab])) OR (Belize[tiab])) OR (Iran[tiab])) OR (Senegal[tiab])) OR (Benin[tiab])) OR (Kenya[tiab])) OR (Melanesia[tiab])) OR (Bhutan[tiab])) OR (Kiribati[tiab])) OR ("Sri Lanka"[tiab])) OR (Bolivia[tiab])) OR (Kyrgyzstan[tiab])) OR (Tanzania[tiab])) OR (Laos[tiab])) OR ("Cabo Verde"[tiab])) OR (Tajikistan[tiab])) OR (Cambodia[tiab])) OR (Lesotho[tiab])) OR ("Timor-Leste"[tiab])) OR (Cameroon[tiab])) OR (Mauritania[tiab])) OR (Tunisia[tiab])) OR (Comoros[tiab])) OR (Micronesia[tiab])) OR (Ukraine[tiab])) OR (Congo[tiab])) OR (Mongolia[tiab])) OR (Uzbekistan[tiab])) OR ("Cote d'Ivoire"[tiab])) OR (Morocco[tiab])) OR (Vanuatu[tiab])) OR (Djibouti[tiab])) OR (Myanmar[tiab])) OR (Vietnam[tiab])) OR (Egypt[tiab])) OR (Nepal[tiab])) OR ("Middle East"[tiab])) OR ("El Salvador"[tiab])) OR (Nicaragua[tiab])) OR (Zambia[tiab])) OR (Eswatini[tiab])) OR (Nigeria[tiab])) OR (Zimbabwe[tiab])) OR (Ghana[tiab])) OR (Pakistan[tiab])) OR (Haiti[tiab])) OR ("Papua New Guinea"[tiab])) OR (((((((((((((((((((((((((((((((((((((((((((("West Bank and Gaza"[tiab]) OR ("Republic of the Gambia"[tiab])) OR ("The Gambia"[tiab])) OR ("North Korea"[tiab])) OR ("North-Korea"[tiab])) OR ("Syrian Arab Republic"[tiab])) OR ("São Tomé and Principe"[tiab])) OR ("Solomon Island*"[tiab])) OR ("Norfolk Island*"[tiab])) OR ("British Solomon Island*"[tiab])) OR (Ceylon[tiab])) OR (Kirghizia[tiab])) OR ("Kyrghyz SSR"[tiab])) OR ("Kyrghiz SSR"[tiab])) OR (Kirgizstan[tiab])) OR ("Kirghiz SSR"[tiab])) OR (Kyrgizstan[tiab])) OR (Kyrgyzstan[tiab])) OR (Kiribati[tiab])) OR (Cabo Verde[tiab])) OR ("Lao People's Democratic Republic"[tiab])) OR (Laos[tiab])) OR ("Timor Leste"[tiab])) OR ("Lao PDR"[tiab])) OR (East Timor[tiab])) OR ("Comor*"[tiab])) OR (Mayotte[tiab])) OR (Brazzaville[tiab])) OR (Palestine[tiab])) OR (Swaziland[tiab])) OR ("Federal Republic of Nigeria"[tiab])) OR (Rhodesia[tiab])) OR ("New Guinea"[tiab])) OR ("East New Guinea"[tiab])) OR ("Uzbek SSR"[tiab])) OR (Uzbek[tiab])) OR ("Côte d'Ivoire"[tiab])) OR ("Ivory Coast"[tiab])) OR ("Cote diIvoire"[tiab])) OR ("New Hebrides"[tiab])) OR ("West bank"[tiab])) OR ("Near east"[tiab])) OR ("Gaza strip"[tiab])) OR (Gaza[tiab]))) OR ((((((((((((((((((((((((((((((((((((((((((((((((((((((((((((((((((((((((((((((("Pakistan"[Mesh]) OR ("Ghana"[Mesh])) OR ("Zimbabwe"[Mesh])) OR ("Nigeria"[Mesh])) OR ("Eswatini"[Mesh])) OR ("Zambia"[Mesh])) OR ("Nicaragua"[Mesh])) OR ("El Salvador"[Mesh])) OR ("Middle East"[Mesh])) OR ("Nepal"[Mesh])) OR ("Egypt"[Mesh])) OR ("Vietnam"[Mesh])) OR ("Myanmar"[Mesh])) OR ("Djibouti"[Mesh])) OR ("Vanuatu"[Mesh])) OR ("Morocco"[Mesh])) OR ("Cote d'Ivoire"[Mesh])) OR ("Uzbekistan"[Mesh])) OR ("Mongolia"[Mesh])) OR ("Ukraine"[Mesh])) OR ("Micronesia"[Mesh])) OR ("Comoros"[Mesh])) OR ("Tunisia"[Mesh])) OR ("Mauritania"[Mesh])) OR ("Cameroon"[Mesh])) OR ("Timor-Leste"[Mesh])) OR ("Lesotho"[Mesh])) OR ("Cambodia"[Mesh])) OR ("Tajikistan"[Mesh])) OR ("Cabo Verde"[Mesh])) OR ("Laos"[Mesh])) OR ("Tanzania"[Mesh])) OR ("Kyrgyzstan"[Mesh])) OR ("Bolivia"[Mesh])) OR ("Sri Lanka"[Mesh])) OR ("Kiribati"[Mesh])) OR ("Bhutan"[Mesh])) OR ("Melanesia"[Mesh])) OR ("Benin"[Mesh])) OR ("Senegal"[Mesh])) OR ("Iran"[Mesh])) OR ("Belize"[Mesh])) OR ("Sao Tome and Principe"[Mesh])) OR ("Indonesia"[Mesh])) OR ("Bangladesh"[Mesh])) OR ("Samoa"[Mesh])) OR ("Algeria"[Mesh])) OR ("Philippines"[Mesh])) OR ("Honduras"[Mesh])) OR ("Angola"[Mesh])) OR ("Yemen"[Mesh])) OR ("Uganda"[Mesh])) OR ("Togo"[Mesh])) OR ("Syria"[Mesh])) OR ("Sudan"[Mesh])) OR ("South Sudan"[Mesh])) OR ("Somalia"[Mesh])) OR ("Sierra Leone"[Mesh])) OR ("Rwanda"[Mesh])) OR ("Niger"[Mesh])) OR ("Mozambique"[Mesh])) OR ("Mali"[Mesh])) OR ("Malawi"[Mesh])) OR ("Madagascar"[Mesh])) OR ("Liberia"[Mesh])) OR ("Democratic People's Republic of Korea"[Mesh])) OR ("Guinea-Bissau"[Mesh])) OR ("Guinea"[Mesh])) OR ("Gambia"[Mesh])) OR ("Ethiopia"[Mesh])) OR ("Eritrea"[Mesh])) OR ("Congo"[Mesh])) OR ("Chad"[Mesh])) OR ("Central African Republic"[Mesh])) OR ("Burundi"[Mesh])) OR ("Afghanistan"[Mesh])) OR ("Burkina Faso"[Mesh])) OR ("Haiti"[Mesh])) OR ("Papua New Guinea"[Mesh]))) OR (((((((((((((((((((("Developing Countries"[Mesh]) OR ("Developing countr*"[tiab])) OR ("Least Developed Countr*"[tiab])) OR ("Less-Developed Countr*"[tiab])) OR ("Less Developed Countr*"[tiab])) OR ("Under-Developed Nation*"[tiab])) OR ("Under Developed Nation*"[tiab])) OR ("Third-World Countr*"[tiab])) OR ("Third World Countr*"[tiab])) OR ("Third-World Nation*"[tiab])) OR ("Third World Nation*"[tiab])) OR ("Under-Developed Countr*"[tiab])) OR ("Under Developed Countr*"[tiab])) OR ("Developing Nation*"[tiab])) OR ("Less-Developed Nation*"[tiab])) OR ("Less Developed Nation*"[tiab])) OR ("middle income country"[tiab])) OR ("low & middle income countr*"[tiab])) OR ("low and middle income countr*"[tiab])) OR ("low middle income countr*"[tiab]))** | 1,360,519 |
| **#6** | **#2 OR #3 OR #4** | 8,953,046 |
| **#7** | **#1 AND #6 AND #5** | 621 |

| ***Search Strategy for Embase*** | | **Date of search:**  23.04.2024 |
| --- | --- | --- |
| **Concept** | **Search Terms** | **Result** |
| **Concept 1: Leptospirosis**  **#1** | **'leptospirosis'/exp OR leptospiroses:ti,ab OR 'leptospira infection':ti,ab OR 'infections leptospira':ti,ab OR 'leptospira infections':ti,ab OR 'leptospira infections':ti,ab OR 'stuttgart disease':ti,ab OR 'mud fever':ti,ab OR 'fevermud':ti,ab OR 'rice-field fever':ti,ab OR 'fever,ruce-field':ti,ab OR 'oryza rice field fever':ti,ab OR 'cane cutter fever':ti,ab OR 'swineherds disease':ti,ab OR 'canicola fever':ti,ab OR 'fever,canicola':ti,ab OR 'leptospira canicola infection':ti,ab OR 'infections, leptospira canicola':ti,ab OR 'leptospira canicola infections':ti,ab OR 'leptospirosis canicola':ti,ab OR 'weil diseas':ti,ab OR 'disease, weil':ti,ab OR 'leptospirosis, icterohemorrhagic':ti,ab OR 'icterohemorrhagic leptospirosis':ti,ab OR 'weils disease':ti,ab OR 'disease, weils':ti,ab OR 'weils disease':ti,ab OR 'jaundice, spirochetal':ti,ab OR 'spirochetal jaundice':ti,ab** | **13,240** |
| **Concept 2.1 : Incidence**  **#2** | **'incidence'/exp OR incidences:ti,ab OR 'secondary attack rate':ti,ab OR 'attack rate, secondary':ti,ab OR 'rate, secondary attack':ti,ab OR 'secondary attack rates':ti,ab OR 'incidence proportion':ti,ab OR 'incidence proportions':ti,ab OR 'proportion, incidence':ti,ab OR 'attack rate':ti,ab OR 'attack rates':ti,ab OR 'rate, attack':ti,ab OR 'cumulative incidence':ti,ab OR 'cumulative incidences':ti,ab OR 'incidence, cumulative':ti,ab OR 'incidence rate':ti,ab OR 'incidence rates':ti,ab OR 'rate, incidence':ti,ab OR 'person-time rate':ti,ab OR 'person time rate':ti,ab** | **804,430** |
| **Concept 2.2: Prevalence**  **#3** | **Prevalence/exp OR Prevalences:ti,ab OR 'Period prevalence':ti,ab OR 'Period prevalences':ti,ab OR 'prevalence period':ti,ab OR 'Point Prevalence':ti,ab OR 'Point Prevalences':ti,ab OR 'prevalence point':ti,ab OR Sero-Prevalence:ti,ab** | **1,050,409** |
| **Concept 2.3: Risk factors**  **#4** | **'Risk Factors'/exp OR 'factor risk':ti,ab OR 'Risk Factor':ti,ab OR 'Social Risk Factors':ti,ab OR 'factors social risk':ti,ab OR 'risk factor social':ti,ab OR 'risk factors social':ti,ab OR 'risk factors*':ti,ab OR 'Social Risk Factor':ti,ab OR 'Health Correlates':ti,ab OR 'correlates health':ti,ab OR 'Population at Risk':ti,ab OR 'Populations at Risk':ti,ab OR 'Risk Scores':ti,ab OR 'Risk Score':ti,ab OR 'score risk':ti,ab OR 'Risk Factor Scores':ti,ab OR 'Risk Factor Scores':ti,ab OR 'Risk Factor Score':ti,ab OR 'score risk factor':ti,ab OR exposure*:ti,ab OR 'Health risk':ti,ab OR vulnerability*:ti,ab OR threat*:ti,ab OR 'risk factor*':ti,ab OR 'Risk Quotient':ti,ab OR determinant*:ti,ab OR predictor*:ti,ab OR cause*:ti,ab OR contributor*:ti,ab OR influence*:ti,ab OR 'associated factor*':ti,ab OR covariates*:ti,ab OR correlates*:ti,ab** | **9,600,712** |
| **Concept 3: LMICs**  **#5** | **(((((((((((((((((((((((((((((((((((((((((((((((((((((((((((((((((((((((((((((((((((“Afghanistan”:ti,ab) OR (“Burkina Faso”:ti,ab)) OR (“Burundi”:ti,ab)) OR (“Central African Republic”:ti,ab)) OR (“Chad”:ti,ab)) OR (“Eritrea”:ti,ab)) OR (“Ethiopia”:ti,ab)) OR (“Gambia”:ti,ab)) OR (“Guinea”:ti,ab)) OR (“Guinea-Bissau”:ti,ab)) OR (“Democratic Peoples Republic of Korea”:ti,ab)) OR (“Liberia”:ti,ab)) OR (“Madagascar”:ti,ab)) OR (“Malawi”:ti,ab)) OR (“Mali”:ti,ab)) OR (“Mozambique”:ti,ab)) OR (“Niger”:ti,ab)) OR (“Rwanda”:ti,ab)) OR (“Sierra Leone”:ti,ab)) OR (“Somalia”:ti,ab)) OR (“South Sudan”:ti,ab)) OR (“Sudan”:ti,ab)) OR (“Syria”:ti,ab)) OR (“Togo”:ti,ab)) OR (“Uganda”:ti,ab)) OR (“Yemen”:ti,ab)) OR (“Angola”:ti,ab)) OR (“Honduras”:ti,ab)) OR (“Philippines”:ti,ab)) OR (“Algeria”:ti,ab)) OR (“India”:ti,ab)) OR (“Samoa”:ti,ab)) OR (“Bangladesh”:ti,ab)) OR (“Indonesia”:ti,ab)) OR (“Sao Tome and Principe”:ti,ab)) OR (“Belize”:ti,ab)) OR (“Iran”:ti,ab)) OR (“Senegal”:ti,ab)) OR (“Benin”:ti,ab)) OR (“Kenya”:ti,ab)) OR (“Melanesia”:ti,ab)) OR (“Bhutan”:ti,ab)) OR (“Kiribati”:ti,ab)) OR (“Sri Lanka”:ti,ab)) OR (“Bolivia”:ti,ab)) OR (“Kyrgyzstan”:ti,ab)) OR (“Tanzania”:ti,ab)) OR (“Laos”:ti,ab)) OR (“Cabo Verde”:ti,ab)) OR (“Tajikistan”:ti,ab)) OR (“Cambodia”:ti,ab)) OR (“Lesotho”:ti,ab)) OR (“Timor-Leste”:ti,ab)) OR (“Cameroon”:ti,ab)) OR (“Mauritania”:ti,ab)) OR (“Tunisia”:ti,ab)) OR (“Comoros”:ti,ab)) OR (“Micronesia”:ti,ab)) OR (“Ukraine”:ti,ab)) OR (“Congo”:ti,ab)) OR (“Mongolia”:ti,ab)) OR (“Uzbekistan”:ti,ab)) OR (“Cote dIvoire”:ti,ab)) OR (“Morocco”:ti,ab)) OR (“Vanuatu”:ti,ab)) OR (“Djibouti”:ti,ab)) OR (“Myanmar”:ti,ab)) OR (“Vietnam”:ti,ab)) OR (“Egypt”:ti,ab)) OR (“Nepal”:ti,ab)) OR (“Middle East”:ti,ab)) OR (“El Salvador”:ti,ab)) OR (“Nicaragua”:ti,ab)) OR (“Zambia”:ti,ab)) OR (“Eswatini”:ti,ab)) OR (“Nigeria”:ti,ab)) OR (“Zimbabwe”:ti,ab)) OR (“Ghana”:ti,ab)) OR (“Pakistan”:ti,ab)) OR (“Haiti”:ti,ab)) OR (“Papua New Guinea”:ti,ab)) OR ((((((((((((((((((((((((((((((((((((((((((((“West Bank and Gaza”:ti,ab) OR (“Republic of the Gambia”:ti,ab)) OR (“The Gambia”:ti,ab)) OR (“North Korea”:ti,ab)) OR (“North-Korea”:ti,ab)) OR (“Syrian Arab Republic”:ti,ab)) OR (“São Tomé and Principe”:ti,ab)) OR (“Solomon Island*”:ti,ab)) OR (“Norfolk Island*”:ti,ab)) OR (“British Solomon Island*”:ti,ab)) OR (“Ceylon”:ti,ab)) OR (“Kirghizia”:ti,ab)) OR (“Kyrghyz SSR”:ti,ab)) OR (“Kyrghiz SSR”:ti,ab)) OR (“Kirgizstan”:ti,ab)) OR (“Kirghiz SSR”:ti,ab)) OR (“Kyrgizstan”:ti,ab)) OR (“Kyrgyzstan”:ti,ab)) OR (“Kiribati”:ti,ab)) OR (“Cabo Verde”:ti,ab)) OR (“Lao Peoples Democratic Republic”:ti,ab)) OR (“Laos”:ti,ab)) OR (“Timor Leste”:ti,ab)) OR (“Lao PDR”:ti,ab)) OR (“East Timor”:ti,ab)) OR (“Comor*”:ti,ab)) OR (“Mayotte”:ti,ab)) OR (“Brazzaville”:ti,ab)) OR (“Palestine”:ti,ab)) OR (“Swaziland”:ti,ab)) OR (“Federal Republic of Nigeria”:ti,ab)) OR (“Rhodesia”:ti,ab)) OR (“New Guinea”:ti,ab)) OR (“East New Guinea”:ti,ab)) OR (“Uzbek SSR”:ti,ab)) OR (“Uzbek”:ti,ab)) OR (“Côte dIvoire”:ti,ab)) OR (“Ivory Coast”:ti,ab)) OR (“Cote diIvoire”:ti,ab)) OR (“New Hebrides”:ti,ab)) OR (“West bank”:ti,ab)) OR (“Near east”:ti,ab)) OR (“Gaza strip”:ti,ab)) OR (“Gaza”:ti,ab))) OR (((((((((((((((((((((((((((((((((((((((((((((((((((((((((((((((((((((((((((((((“Pakistan”/exp) OR (“Ghana”/exp)) OR (“Zimbabwe”/exp)) OR (“Nigeria”/exp)) OR (“Eswatini”/exp)) OR (“Zambia”/exp)) OR (“Nicaragua”/exp)) OR (“El Salvador”/exp)) OR (“Middle East”/exp)) OR (“Nepal”/exp)) OR (“Egypt”/exp)) OR (“Vietnam”/exp)) OR (“Myanmar”/exp)) OR (“Djibouti”/exp)) OR (“Vanuatu”/exp)) OR (“Morocco”/exp)) OR (“Cote dIvoire”/exp)) OR (“Uzbekistan”/exp)) OR (“Mongolia”/exp)) OR (“Ukraine”/exp)) OR (“Micronesia”/exp)) OR (“Comoros”/exp)) OR (“Tunisia”/exp)) OR (“Mauritania”/exp)) OR (“Cameroon”/exp)) OR (“Timor-Leste”/exp)) OR (“Lesotho”/exp)) OR (“Cambodia”/exp)) OR (“Tajikistan”/exp)) OR (“Cabo Verde“/exp)) OR (“Laos”/exp)) OR (“Tanzania”/exp)) OR (“Kyrgyzstan”/exp)) OR (“Bolivia”/exp)) OR (“Sri Lanka“/exp)) OR (“Kiribati”/exp)) OR (“Bhutan”/exp)) OR (“Melanesia”/exp)) OR (“Benin”/exp)) OR (“Senegal”/exp)) OR (“Iran”/exp)) OR (“Belize”/exp)) OR (“Sao Tome and Principe“/exp)) OR (“Indonesia”/exp)) OR (“Bangladesh”/exp)) OR (“Samoa”/exp)) OR (“Algeria”/exp)) OR (“Philippines”/exp)) OR (“Honduras”/exp)) OR (“Angola”/exp)) OR (“Yemen”/exp)) OR (“Uganda”/exp)) OR (“Togo”/exp)) OR (“Syria”/exp)) OR (“Sudan”/exp)) OR (“South Sudan“/exp)) OR (“Somalia”/exp)) OR (“Sierra Leone”/exp)) OR (“Rwanda”/exp)) OR (“Niger”/exp)) OR (“Mozambique”/exp)) OR (“Mali”/exp)) OR (“Malawi”/exp)) OR (“Madagascar”/exp)) OR (“Liberia”/exp)) OR (“Democratic Peoples Republic of Korea“/exp)) OR (“Guinea-Bissau”/exp)) OR (“Guinea”/exp)) OR (“Gambia”/exp)) OR (“Ethiopia”/exp)) OR (“Eritrea”/exp)) OR (“Congo”/exp)) OR (“Chad”/exp)) OR (“Central African Republic”/exp)) OR (“Burundi”/exp)) OR (“Afghanistan”/exp)) OR (“Burkina Faso“/exp)) OR (“Haiti”/exp)) OR (“Papua New Guinea“/exp))) OR ((((((((((((((((((((“Developing Countries”/exp) OR (“Developing countr*”:ti,ab)) OR (“Least Developed Countr*”:ti,ab)) OR (“Less-Developed Countr*”:ti,ab)) OR (“Less Developed Countr*”:ti,ab)) OR (“Under-Developed Nation*”:ti,ab)) OR (“Under Developed Nation*”:ti,ab)) OR (“Third-World Countr*”:ti,ab)) OR (“Third World Countr*”:ti,ab)) OR (“Third-World Nation*”:ti,ab)) OR (“Third World Nation*”:ti,ab)) OR (“Under-Developed Countr*”:ti,ab)) OR (“Under Developed Countr*”:ti,ab)) OR (“Developing Nation*”:ti,ab)) OR (“Less-Developed Nation*”:ti,ab)) OR (“Less Developed Nation*”:ti,ab)) OR (“middle income country”:ti,ab)) OR (“low & middle income countr*”:ti,ab)) OR (“low and middle income countr*”:ti,ab)) OR (“low middle income countr*”:ti,ab))** | **1,806,844** |
| **#6** | **#2 OR #3 OR #4** | **1,0546,701** |
| **#7** | **#1 AND #6 AND #5** | **1,141** |

| ***Search Strategy for CINAHL (Ebsco)*** | | **Date of search:**  23.04.2024 |
| --- | --- | --- |
| **Concept** | **Search Terms** | **Result** |
| **Concept 1: Leptospirosis**  **#1** | **((((((((((((((((((((((((((((((((((MH Leptospirosis+)) OR ((TI Leptospiroses OR AB Leptospiroses))) OR ((TI "Leptospira Infection" OR AB "Leptospira Infection"))) OR ((TI "Infection, Leptospira" OR AB "Infection, Leptospira"))) OR ((TI "Infections, Leptospira" OR AB "Infections, Leptospira"))) OR ((TI "Leptospira Infections" OR AB "Leptospira Infections"))) OR ((TI "Stuttgart Disease" OR AB "Stuttgart Disease"))) OR ((TI "Mud Fever" OR AB "Mud Fever"))) OR ((TI "Fever, Mud" OR AB "Fever, Mud"))) OR ((TI "Rice-Field Fever" OR AB "Rice-Field Fever"))) OR ((TI "Fever, Rice-Field" OR AB "Fever, Rice-Field"))) OR (((MH oryza+) OR oryza OR rice) AND (TI "field fever" OR AB "field fever"))) OR (Cane-Cutter AND (TI Fever OR AB Fever))) OR ((((MH canes+) OR canes OR cane) AND (cutter OR cutters)) AND (TI Fever OR AB Fever))) OR (((MH fever+) OR fever OR fevers) AND (TI Cane-Cutter OR AB Cane-Cutter))) OR ((TI "Swineherd's Disease" OR AB "Swineherd's Disease"))) OR ((TI "Swineherd's Diseases" OR AB "Swineherd's Diseases"))) OR ((TI "Canicola Fever" OR AB "Canicola Fever"))) OR ((TI "Fever, Canicola" OR AB "Fever, Canicola"))) OR ((TI "Leptospira Canicola Infection" OR AB "Leptospira Canicola Infection"))) OR ((TI "Infections, Leptospira Canicola" OR AB "Infections, Leptospira Canicola"))) OR ((TI "Leptospira Canicola Infections" OR AB "Leptospira Canicola Infections"))) OR ((TI "Leptospirosis Canicola" OR AB "Leptospirosis Canicola"))) OR ((TI "leptospirosis canicola" OR AB "leptospirosis canicola"))) OR ((MH "Weil Disease+"))) OR ((TI "Disease, Weil" OR AB "Disease, Weil"))) OR ((TI "Leptospirosis, Icterohemorrhagic" OR AB "Leptospirosis, Icterohemorrhagic"))) OR ((TI "Icterohemorrhagic Leptospirosis" OR AB "Icterohemorrhagic Leptospirosis"))) OR ((TI "Weil's Disease" OR AB "Weil's Disease"))) OR ((TI "Disease, Weil's" OR AB "Disease, Weil's"))) OR ((TI "Weils Disease" OR AB "Weils Disease"))) OR ((TI "Jaundice, Spirochetal" OR AB "Jaundice, Spirochetal"))) OR ((TI "Spirochetal Jaundice" OR AB "Spirochetal Jaundice")))** | 477 |
| **Concept 2.1 : Incidence**  **#2** | **(((((((((((((((((((((((((((MH Incidence+)) OR ((TI "Rate, Person-time" OR AB "Rate, Person-time"))) OR ((TI "Person-time Rates" OR AB "Person-time Rates"))) OR ((TI "Person time Rate"" OR AB "Person time Rate""))) OR ((TI "Person-time Rate" OR AB "Person-time Rate"))) OR ((TI "Rate, Incidence" OR AB "Rate, Incidence"))) OR (OR (TI "Incidence Rates" OR AB "Incidence Rates"))) OR ((TI "Incidence Rate" OR AB "Incidence Rate"))) OR ((TI "Incidence, Cumulative" OR AB "Incidence, Cumulative"))) OR ((TI "Cumulative Incidences" OR AB "Cumulative Incidences"))) OR ((TI "Cumulative Incidence" OR AB "Cumulative Incidence"))) OR ((TI "Rate, Attack" OR AB "Rate, Attack"))) OR ((TI "Attack Rates" OR AB "Attack Rates"))) OR ((TI "Attack Rate" OR AB "Attack Rate"))) OR ((TI "Proportion, Incidence" OR AB "Proportion, Incidence"))) OR ((TI "Incidence Proportions" OR AB "Incidence Proportions"))) OR ((TI "Incidence Proportion" OR AB "Incidence Proportion"))) OR ((TI "Secondary Attack Rates" OR AB "Secondary Attack Rates"))) OR ((TI "Rate, Secondary Attack" OR AB "Rate, Secondary Attack"))) OR ((TI "Rate, Secondary Attack" OR AB "Rate, Secondary Attack"))) OR ((TI "Attack Rate, Secondary" OR AB "Attack Rate, Secondary"))) OR ((TI "Secondary Attack Rate" OR AB "Secondary Attack Rate"))) OR ((TI Incidences OR AB Incidences))) OR ((TI Incidence OR AB Incidence))) OR ((TI distribution* OR AB distribution*))) OR ((TI Epidemiology OR AB Epidemiology))) OR ((TI "incidence rate*" OR AB "incidence rate*"))** | 333,484 |
| **Concept 2.2: Prevalence**  **#3** | **(((((((((MH Prevalence+)) OR ((TI Prevalences OR AB Prevalences))) OR ((TI "Period prevalence" OR AB "Period prevalence"))) OR ((TI "Period prevalences" OR AB "Period prevalences"))) OR ((TI "prevalence period" OR AB "prevalence period"))) OR ((TI "Point Prevalence" OR AB "Point Prevalence"))) OR ((TI "Point Prevalences" OR AB "Point Prevalences"))) OR ((TI "prevalence point" OR AB "prevalence point"))) OR ((TI Sero-Prevalence OR AB Sero-Prevalence))** | 254,814 |
| **Concept 2.3: Risk factors**  **#4** | **((((((((((((((((((((((((((((((((((((MH "Risk Factors+")) OR ((TI "Factor, Risk" OR AB "Factor, Risk"))) OR ((TI "Risk Factor" OR AB "Risk Factor"))) OR ((TI "Social Risk Factors" OR AB "Social Risk Factors"))) OR ((TI "Factor, Social Risk" OR AB "Factor, Social Risk"))) OR ((TI "Factor, Social Risk" OR AB "Factor, Social Risk"))) OR ((TI "Factors, Social Risk" OR AB "Factors, Social Risk"))) OR ((TI "Risk Factor, Social" OR AB "Risk Factor, Social"))) OR ((TI "Risk Factors, Social" OR AB "Risk Factors, Social"))) OR ((TI "risk factors*" OR AB "risk factors*"))) OR ((TI "Social Risk Factor" OR AB "Social Risk Factor"))) OR ((TI "Health Correlates" OR AB "Health Correlates"))) OR ((TI "Correlates, Health" OR AB "Correlates, Health"))) OR ((TI "Population at Risk" OR AB "Population at Risk"))) OR ((TI "Populations at Risk" OR AB "Populations at Risk"))) OR ((TI "Risk Scores" OR AB "Risk Scores"))) OR ((TI "Risk Score" OR AB "Risk Score"))) OR ((TI "Score, Risk" OR AB "Score, Risk"))) OR ((TI "Risk Factor Scores" OR AB "Risk Factor Scores"))) OR ((TI "Risk Factor Scores" OR AB "Risk Factor Scores"))) OR ((TI "Risk Factor Score" OR AB "Risk Factor Score"))) OR ((TI "Score, Risk Factor" OR AB "Score, Risk Factor"))) OR ((TI Exposure* OR AB Exposure*))) OR ((TI "Health risk" OR AB "Health risk"))) OR ((TI Vulnerability* OR AB Vulnerability*))) OR ((TI Threat* OR AB Threat*))) OR ((TI "risk factor*" OR AB "risk factor*"))) OR ((TI "Risk Quotient" OR AB "Risk Quotient"))) OR ((TI Determinant* OR AB Determinant*))) OR ((TI Predictor* OR AB Predictor*))) OR ((TI Cause* OR AB Cause*))) OR ((TI Contributor* OR AB Contributor*))) OR ((TI Influence* OR AB Influence*))) OR ((TI "Associated factor*" OR AB "Associated factor*"))) OR ((TI Covariates* OR AB Covariates*))) OR ((TI Correlates* OR AB Correlates*))** | 1,304,947 |
| **Concept 3: LMICs**  **#5** | **((((((((((((((((((((((((((((((((((((((((((((((((((((((((((((((((((((((((((((((((((((TI Afghanistan OR AB Afghanistan)) OR ((TI "Burkina Faso" OR AB "Burkina Faso"))) OR ((TI Burundi OR AB Burundi))) OR ((TI "Central African Republic" OR AB "Central African Republic"))) OR ((TI Chad OR AB Chad))) OR ((TI Eritrea OR AB Eritrea))) OR ((TI Ethiopia OR AB Ethiopia))) OR ((TI Gambia OR AB Gambia))) OR ((TI Guinea OR AB Guinea))) OR ((TI Guinea-Bissau OR AB Guinea-Bissau))) OR ((TI "Democratic People's Republic of Korea" OR AB "Democratic People's Republic of Korea"))) OR ((TI Liberia OR AB Liberia))) OR ((TI Madagascar OR AB Madagascar))) OR ((TI Malawi OR AB Malawi))) OR ((TI Mali OR AB Mali))) OR ((TI Mozambique OR AB Mozambique))) OR ((TI Niger OR AB Niger))) OR ((TI Rwanda OR AB Rwanda))) OR ((TI "Sierra Leone" OR AB "Sierra Leone"))) OR ((TI Somalia OR AB Somalia))) OR ((TI "South Sudan" OR AB "South Sudan"))) OR ((TI Sudan OR AB Sudan))) OR ((TI Syria OR AB Syria))) OR ((TI Togo OR AB Togo))) OR ((TI Uganda OR AB Uganda))) OR ((TI Yemen OR AB Yemen))) OR ((TI Angola OR AB Angola))) OR ((TI Honduras OR AB Honduras))) OR ((TI Philippines OR AB Philippines))) OR ((TI Algeria OR AB Algeria))) OR ((TI India OR AB India))) OR ((TI Samoa OR AB Samoa))) OR ((TI Bangladesh OR AB Bangladesh))) OR ((TI Indonesia OR AB Indonesia))) OR ((TI "Sao Tome and Principe" OR AB "Sao Tome and Principe"))) OR ((TI Belize OR AB Belize))) OR ((TI Iran OR AB Iran))) OR ((TI Senegal OR AB Senegal))) OR ((TI Benin OR AB Benin))) OR ((TI Kenya OR AB Kenya))) OR ((TI Melanesia OR AB Melanesia))) OR ((TI Bhutan OR AB Bhutan))) OR ((TI Kiribati OR AB Kiribati))) OR ((TI "Sri Lanka" OR AB "Sri Lanka"))) OR ((TI Bolivia OR AB Bolivia))) OR ((TI Kyrgyzstan OR AB Kyrgyzstan))) OR ((TI Tanzania OR AB Tanzania))) OR ((TI Laos OR AB Laos))) OR ((TI "Cabo Verde" OR AB "Cabo Verde"))) OR ((TI Tajikistan OR AB Tajikistan))) OR ((TI Cambodia OR AB Cambodia))) OR ((TI Lesotho OR AB Lesotho))) OR ((TI Timor-Leste OR AB Timor-Leste))) OR ((TI Cameroon OR AB Cameroon))) OR ((TI Mauritania OR AB Mauritania))) OR ((TI Tunisia OR AB Tunisia))) OR ((TI Comoros OR AB Comoros))) OR ((TI Micronesia OR AB Micronesia))) OR ((TI Ukraine OR AB Ukraine))) OR ((TI Congo OR AB Congo))) OR ((TI Mongolia OR AB Mongolia))) OR ((TI Uzbekistan OR AB Uzbekistan))) OR ((TI "Cote d'Ivoire" OR AB "Cote d'Ivoire"))) OR ((TI Morocco OR AB Morocco))) OR ((TI Vanuatu OR AB Vanuatu))) OR ((TI Djibouti OR AB Djibouti))) OR ((TI Myanmar OR AB Myanmar))) OR ((TI Vietnam OR AB Vietnam))) OR ((TI Egypt OR AB Egypt))) OR ((TI Nepal OR AB Nepal))) OR ((TI "Middle East" OR AB "Middle East"))) OR ((TI "El Salvador" OR AB "El Salvador"))) OR ((TI Nicaragua OR AB Nicaragua))) OR ((TI Zambia OR AB Zambia))) OR ((TI Eswatini OR AB Eswatini))) OR ((TI Nigeria OR AB Nigeria))) OR ((TI Zimbabwe OR AB Zimbabwe))) OR ((TI Ghana OR AB Ghana))) OR ((TI Pakistan OR AB Pakistan))) OR ((TI Haiti OR AB Haiti))) OR ((TI "Papua New Guinea" OR AB "Papua New Guinea"))) OR (((((((((((((((((((((((((((((((((((((((((((((TI "West Bank and Gaza" OR AB "West Bank and Gaza")) OR ((TI "Republic of the Gambia" OR AB "Republic of the Gambia"))) OR ((TI "The Gambia" OR AB "The Gambia"))) OR ((TI "North Korea" OR AB "North Korea"))) OR ((TI North-Korea OR AB North-Korea))) OR ((TI "Syrian Arab Republic" OR AB "Syrian Arab Republic"))) OR ((TI "São Tomé and Principe" OR AB "São Tomé and Principe"))) OR ((TI "Solomon Island*" OR AB "Solomon Island*"))) OR ((TI "Norfolk Island*" OR AB "Norfolk Island*"))) OR ((TI "British Solomon Island*" OR AB "British Solomon Island*"))) OR ((TI Ceylon OR AB Ceylon))) OR ((TI Kirghizia OR AB Kirghizia))) OR ((TI "Kyrghyz SSR" OR AB "Kyrghyz SSR"))) OR ((TI "Kyrghiz SSR" OR AB "Kyrghiz SSR"))) OR ((TI Kirgizstan OR AB Kirgizstan))) OR ((TI "Kirghiz SSR" OR AB "Kirghiz SSR"))) OR ((TI Kyrgizstan OR AB Kyrgizstan))) OR ((TI Kyrgyzstan OR AB Kyrgyzstan))) OR ((TI Kiribati OR AB Kiribati))) OR ((TI "Cabo Verde" OR AB "Cabo Verde"))) OR ((TI "Lao People's Democratic Republic" OR AB "Lao People's Democratic Republic"))) OR ((TI Laos OR AB Laos))) OR ((TI "Timor Leste" OR AB "Timor Leste"))) OR ((TI "Lao PDR" OR AB "Lao PDR"))) OR ((TI "East Timor" OR AB "East Timor"))) OR ((TI Comor* OR AB Comor*))) OR ((TI Mayotte OR AB Mayotte))) OR ((TI Brazzaville OR AB Brazzaville))) OR ((TI Palestine OR AB Palestine))) OR ((TI Swaziland OR AB Swaziland))) OR ((TI "Federal Republic of Nigeria" OR AB "Federal Republic of Nigeria"))) OR ((TI Rhodesia OR AB Rhodesia))) OR ((TI "New Guinea" OR AB "New Guinea"))) OR ((TI "East New Guinea" OR AB "East New Guinea"))) OR ((TI "Uzbek SSR" OR AB "Uzbek SSR"))) OR ((TI Uzbek OR AB Uzbek))) OR ((TI "Côte d'Ivoire" OR AB "Côte d'Ivoire"))) OR ((TI "Ivory Coast" OR AB "Ivory Coast"))) OR ((TI "Cote diIvoire" OR AB "Cote diIvoire"))) OR ((TI "New Hebrides" OR AB "New Hebrides"))) OR ((TI "West bank" OR AB "West bank"))) OR ((TI "Near east" OR AB "Near east"))) OR ((TI "Gaza strip" OR AB "Gaza strip"))) OR ((TI Gaza OR AB Gaza)))) OR ((((((((((((((((((((((((((((((((((((((((((((((((((((((((((((((((((((((((((((((((MH Pakistan+)) OR ((MH Ghana+))) OR ((MH Zimbabwe+))) OR ((MH Nigeria+))) OR ((MH Eswatini+))) OR ((MH Zambia+))) OR ((MH Nicaragua+))) OR ((MH "El Salvador+"))) OR ((MH "Middle East+"))) OR ((MH Nepal+))) OR ((MH Egypt+))) OR ((MH Vietnam+))) OR ((MH Myanmar+))) OR ((MH Djibouti+))) OR ((MH Vanuatu+))) OR ((MH Morocco+))) OR ((MH "Cote d'Ivoire+"))) OR ((MH Uzbekistan+))) OR ((MH Mongolia+))) OR ((MH Ukraine+))) OR ((MH Micronesia+))) OR ((MH Comoros+))) OR ((MH Tunisia+))) OR ((MH Mauritania+))) OR ((MH Cameroon+))) OR ((MH Timor-Leste+))) OR ((MH Lesotho+))) OR ((MH Cambodia+))) OR ((MH Tajikistan+))) OR ((MH "Cabo Verde+"))) OR ((MH Laos+))) OR ((MH Tanzania+))) OR ((MH Kyrgyzstan+))) OR ((MH Bolivia+))) OR ((MH "Sri Lanka+"))) OR ((MH Kiribati+))) OR ((MH Bhutan+))) OR ((MH Melanesia+))) OR ((MH Benin+))) OR ((MH Senegal+))) OR ((MH Iran+))) OR ((MH Belize+))) OR ((MH "Sao Tome and Principe+"))) OR ((MH Indonesia+))) OR ((MH Bangladesh+))) OR ((MH Samoa+))) OR ((MH Algeria+))) OR ((MH Philippines+))) OR ((MH Honduras+))) OR ((MH Angola+))) OR ((MH Yemen+))) OR ((MH Uganda+))) OR ((MH Togo+))) OR ((MH Syria+))) OR ((MH Sudan+))) OR ((MH "South Sudan+"))) OR ((MH Somalia+))) OR ((MH "Sierra Leone+"))) OR ((MH Rwanda+))) OR ((MH Niger+))) OR ((MH Mozambique+))) OR ((MH Mali+))) OR ((MH Malawi+))) OR ((MH Madagascar+))) OR ((MH Liberia+))) OR ((MH "Democratic People's Republic of Korea+"))) OR ((MH Guinea-Bissau+))) OR ((MH Guinea+))) OR ((MH Gambia+))) OR ((MH Ethiopia+))) OR ((MH Eritrea+))) OR ((MH Congo+))) OR ((MH Chad+))) OR ((MH "Central African Republic+"))) OR ((MH Burundi+))) OR ((MH Afghanistan+))) OR ((MH "Burkina Faso+"))) OR ((MH Haiti+))) OR ((MH "Papua New Guinea+")))) OR (((((((((((((((((((((MH "Developing Countries+")) OR ((TI "Developing countr*" OR AB "Developing countr*"))) OR ((TI "Least Developed Countr*" OR AB "Least Developed Countr*"))) OR ((TI "Less-Developed Countr*" OR AB "Less-Developed Countr*"))) OR ((TI "Less Developed Countr*" OR AB "Less Developed Countr*"))) OR ((TI "Under-Developed Nation*" OR AB "Under-Developed Nation*"))) OR ((TI "Under Developed Nation*" OR AB "Under Developed Nation*"))) OR ((TI "Third-World Countr*" OR AB "Third-World Countr*"))) OR ((TI "Third World Countr*" OR AB "Third World Countr*"))) OR ((TI "Third-World Nation*" OR AB "Third-World Nation*"))) OR ((TI "Third World Nation*" OR AB "Third World Nation*"))) OR ((TI "Under-Developed Countr*" OR AB "Under-Developed Countr*"))) OR ((TI "Under Developed Countr*" OR AB "Under Developed Countr*"))) OR ((TI "Developing Nation*" OR AB "Developing Nation*"))) OR ((TI "Less-Developed Nation*" OR AB "Less-Developed Nation*"))) OR ((TI "Less Developed Nation*" OR AB "Less Developed Nation*"))) OR ((TI "middle income country" OR AB "middle income country"))) OR ((TI "low & middle income countr*" OR AB "low & middle income countr*"))) OR ((TI "low and middle income countr*" OR AB "low and middle income countr*"))) OR ((TI "low middle income countr*" OR AB "low middle income countr*")))** | 365,619 |
| **#6** | **#2 OR #3 OR #4** | 16,76,245 |
| **#7** | **#1 AND #6 AND #5** | 93 |

**Search Methodology:**

A comprehensive literature search was conducted to identify studies reporting the incidence, seroprevalence, and risk factors of leptospirosis in low- and middle-income countries (LMICs). The search strategy was developed around five key concepts:

a. Leptospirosis,
b. Incidence,
c. Prevalence,
d. Risk factors, and
e. Low- and Middle-Income Countries (LMICs).

The initial search strategy was developed for MEDLINE using Medical Subject Headings (MeSH), relevant keywords and entry terms identified from previously published literature, and appropriate Boolean operators (AND, OR). The strategy was subsequently adapted for EMBASE and CINAHL using the corresponding controlled vocabulary, including ***Emtree terms*** and ***CINAHL Headings***, as appropriate. The complete database-specific search strategies are provided in this supplementary file. In addition, the first ten pages of Google Scholar search results were screened to identify potentially relevant literature not retrieved through the electronic databases.

The databases were searched and records were downloaded for primary screening on 23 April 2024. The search was restricted to English-language publications. No additional filters based on publication year or study design were applied. The detailed inclusion and exclusion criteria can be found in the table below.

|  | **Inclusion criteria** | **Exclusion criteria** |
| --- | --- | --- |
| **Population** | Studies reporting data on individuals diagnosed with leptospirosis or examining leptospirosis at the population level | Studies reporting incidence or seroprevalence data exclusively on animals |
| **Study setting** | Studies conducted in low- and middle-income countries (LMICs), based on the World Bank classification (2024) | Countries other than LMICs were excluded, irrespective of their content |
| **Outcomes** | Studies reporting incidence, prevalence, or risk factors of leptospirosis. If the studies included numerator, denominator, and other relevant information to calculate incidence, like time period - those articles were also included. | Besides incidence, prevalence and risk factors, studies that failed to provide the time period necessary for calculating incidence or prevalence |
| **Study design / evidence type** | Studies providing observational data on leptospirosis incidence, seroprevalence, or risk factors | Narrative reviews, scoping reviews, conference abstracts, and opinion articles |
| **Population at risk** |  | Studies that did not specify the population at risk |
| **Language** | Studies published only in English | Language other than English were not included, since translation using software or AI may not be exactly similar to what the authors wanted to describe |
