# Supplementary material for: Unmasking the burden and risk factors of leptospirosis in low- and middle-income countries: a systematic review and meta-analysis
Source: One Health. 2026 Oct 24;23:101632. doi: 10.1016/j.onehlt.2026.101632 (PMC13742084; doi:10.1016/j.onehlt.2026.101632)
Supplement: Supplementary file 3 [file mmc4.docx]

**Methods followed for the Meta analysis**

25 studies (Incidence: 5 studies and Prevalence: 20 studies) were found eligible for data extraction. We prepared two data extraction sheets (Supplementary file 3) – one each for Incidence and Prevalence. Once the relevant information was extracted, the data was taken to statistical software STATA v18.0. We used the *meta* command to conduct the meta-analysis, making forest plots, addressing heterogeneity among the studies and further iteration required.

A. **Meta Analysis of incidence:**

In the data extraction sheet, we extracted the *number of leptospirosis cases* found during the study period, *population size at risk* and *study duration*. We estimated the *incidence rate* (cases per 1000 population per year) using the formula below:

$$\frac{Number of new cases}{Population size at risk*Duration of the study}\times1000$$

Following this, the 95% confidence interval was computed.

We began with estimating the pooled incidence for all the 5 studies, where we found a high heterogeneity (I^2^) (Figure S1).

**Figure S1: Pooled incidence rate (per 1000 per year) including all five studies**

Due to high I^2^ we performed sensitivity analysis using leave-one-out (LOO) method (Figure S2).

**Figure S2: Sensitivity analysis using leave-one-out method**

It was evident that the level of heterogeneity remained unaffected even after applying leave-one-out (LOO) methos. However, there was a significant change in the estimate from 130.15 cases per 1000 per year to 47.28 cases per 1000 per year cases after omitting a study by Unnikrishnan et al., 2005 (Figure S1 and S2).

Therefore, we omitted this influential study from meta-analysis and estimated the pooled incidence rate from the rest four research (Figure S3).

**Figure S3: Pooled incidence rate (cases per 1000 per year) including four studies (removing Unnikrishnan et al., 2005)**

Again, we conducted the sensitivity analysis on these 4 studies using the leave-one-out method. The second LOO helped to evaluate the robustness of the reduced model and ensured whether another study becomes influential once the dominant outlier is removed. After doing so, we identified another influencing study by Marcovych et al., 2016, where the heterogeneity attenuated from 99.24% to 83.1% after omitting this study. (Figure S4, Supplementary Table 1). The description of I^2^ values has been presented in the Supplementary Table 1 below:

**Supplementary Table 1: Combination of studies used to estimate the pooled incidence rate after sensitivity analysis.**

| **Sl No** | **Studies Included (Authors & Year)** | **I² (%)** |
| --- | --- | --- |
| **1** | Banukumar et al., 2016; Markovych et al., 2019; Cunha & Costa, 2022 | 99.65 |
| **2** | Bigg et al., 2013; Markovych et al., 2019; Cunha & Costa, 2022 | 99.19 |
| **3** | Bigg et al., 2013; Banukumar et al., 2016; Cunha & Costa, 2022 | 83.12 |
| **4** | Bigg et al., 2013; Banukumar et al., 2016; Markovych et al., 2019 | 96.33 |

**Figure S4: Sensitivity analysis using leave-one-out method (removing Unnikrishnan et al., 2005)**

Hence, we estimated the pooled incidence rate for three studies after omitting two influential studies which has been presented as figure 3 in the manuscript.

B. **Meta Analysis of prevalence:**

During the data extraction phase we identified number of cases, the population size (from where the cases were drawn cross-sectionally), study setting (if the survey included the whole community; only a part of the community, which are considered as high-risk community – more susceptible to the leptospira infection; or patients coming to health facilities and got diagnosed with leptospirosis), diagnostic method used – ELISA or MAT – to identify the antibody (IgG, IgM or both) against the pathogen of interest.

We started with calculating the prevalence as percentage for each of the 20 studies using the formula below:

Prevalence = $\frac{No. of cases}{total population}\times100$

Following that, the standard error for prevalence in percentage was calculated for all the studies.

Since, the cross-sectional studies included are community seroprevalence, facility based and high risk occupational seroprevalence with varying diagnostic methods (MAT/ELISA), these made pooled prevalence difficult to interpret. Therefore, taking all these into account, we pooled the estimates accordingly for “1. community-based”, “2. facility-based” and “3. high-risk population” (Figure 4).

1. ***Community-based***

A total of 9 studies were pooled for the estimation of community-based prevalence (Figure 4). Here, the heterogeneity was considerably high, so, sensitivity analysis using LOO method was performed which hardly made any difference in terms of pooled estimate and heterogeneity (Figure S5). Therefore, a subgroup analysis was planned where *community* was stratified based on diagnostic methods (ELISA vs MAT). Upon stratification also heterogeneity remained high (Figure S6).

**Figure S5: Sensitivity analysis using leave-one-out method for community-based studies (n=9)**

**Figure S6: Subgroup analysis stratified by diagnostic methods [ELISA (n=2) and MAT (n=7)]**

2. ***Facility-based***

A total of 7 studies were pooled for the estimation of facility-based prevalence (Figure 4). Here, the heterogeneity was considerably high, so, sensitivity analysis using LOO method was performed which hardly made any difference in terms of pooled estimate and heterogeneity (Figure S7). Therefore, a subgroup analysis was planned where facility was stratified based on diagnostics test (ELISA vs. MAT). Upon stratification also heterogeneity remained high (Figure S8).

**Figure S7: Sensitivity analysis using leave-one-out method for facility-based studies, (n=7)**

**Figure S8: Subgroup analysis stratified by diagnostic methods [ELISA (n=4) and MAT (n=3)]**

3. ***High-risk population***

Four studies focused on the high-risk populations, which included:

a. residents of ten sewage drainage basins in Brazil (Dias et al., 2007)
b. hunters and their families, butchers and slaughterhouse workers, and healthcare workers from Iran (Esmaeili et al., 2016)
c. rice cultivators, fish farming workers, livestock owner from northern Egypt (Hegazy et al., 2021)
d. low-income agricultural communities from Rwanda (Ntabanganyimana et al., 2021)

The prevalence of leptospirosis in high-risk group was 18.78% (6.15%, 31.41). Though marginally less than other two groups, the heterogeneity was persistent in the sensitivity analysis (Figure S9). A separate subgroup analysis for high-risk populations was not performed because the small number of studies[1].

**Figure S9: Sensitivity analysis using leave-one-out method for high-risk population-based studies, (n=4)**

Given persistent heterogeneity, an exploratory meta-regression was performed to examine study-level predictors. Most variables did not account for heterogeneity. Country explained a small proportion (~5%) of between-study variance, but substantial residual heterogeneity remained unexplained. Moreover, the meta regression was performed on 13 studies combining community and high-risk populations. These were combined as they represent comparable population-based contexts, improving statistical stability and interpretability of meta-regression estimates. Facility-based studies were excluded due to insufficient representation for reliable modelling[1].

**Supplementary Table 2: Meta-regression assessing study-level predictors explaining between-study heterogeneity**

| **Predictor Variable** | **β Coefficient (95% CI)** | **R² (%)** | **I² (%)** | **τ²** |
| --- | --- | --- | --- | --- |
| Diagnostic method (ELISA/MAT) | –0.35 (–1.85, 1.15) | 0.00 | 99.37 | 1.586 |
| Setting | –0.44 (–1.93, 1.05) | 0.00 | 99.35 | 1.568 |
| Country | 0.16 (–0.07, 0.38) | 5.28 | 99.26 | 1.398 |
| Year | –0.05 (–0.14, 0.04) | 0.94 | 99.28 | 1.462 |

**Figure S10: Subgroup analysis stratified by Geographic location [Asia (n=16)]**

The forest plot shows that leptospirosis positivity was generally higher in African studies than in Asian studies. The pooled positivity was 24.87% (95% CI: 15.77–33.96) in Asia and 25.75% (95% CI: 6.51–45.00) in Africa. Both regions showed very high heterogeneity (I² = 99.60% and 98.06%, respectively), indicating substantial variation across studies. Overall, the pooled estimates were statistically significant in both regions.

**Figure S11: Subgroup analysis stratified by study settings (Urban/Rural)**

Leptospirosis positivity was 22.68% (95% CI: 11.12–34.24) in rural-only studies, compared with 26.76% (95% CI: 14.34–39.17) in urban-only studies. Studies including both rural and urban settings showed a pooled positivity of 23.34% (95% CI: 7.98–38.70). Heterogeneity was extremely high across all three groups (I² = 98.52–99.77%), indicating substantial variation between studies.

**Figure S12: Subgroup analysis stratified by study period (Studies conducted before 2015 vs studies commenced in 2015 or later**

Leptospirosis positivity was higher in studies conducted before 2015, with a pooled prevalence of 28.74% (95% CI: 17.87–39.61), compared with 19.98% (95% CI: 9.15–30.81) in studies conducted during 2015 or later. Both groups showed very high heterogeneity (I² = 99.22% and 99.61%, respectively).

**Certainty of Evidence Assessment for incidence of leptospirosis in LMICs**

*Risk of bias* was judged to be serious, as all three included studies were classified as having unclear overall risk of bias. Methodological concerns were particularly evident in the assessment and adjustment of confounding factors, with Banukumar et al. (2016)a demonstrating high risk in both domains. Additional uncertainties were identified in participant recruitment, baseline outcome status, and follow-up completeness.

*Inconsistency* was considered serious owing to substantial residual heterogeneity (I² = 83.12%), despite the exclusion of two influential studies during sensitivity analysis.

*Indirectness* was considered serious because the incidence evidence was restricted to studies conducted in Brazil, India, and Tanzania, limiting its applicability to the wider LMIC population.

*Imprecision* was provisionally judged to be serious because the confidence interval encompassed a potentially meaningful range of incidence estimates (42.10-71.62 cases per 1,000 population per year).

*Publication bias* could not be reliably assessed statistically because only three studies contributed to the final meta-analysis, rendering funnel-plot asymmetry assessment and Egger's regression test unsuitable.

Overall, the proposed very low certainty rating indicates limited confidence in the pooled incidence estimate. These findings should be interpreted cautiously, particularly when extrapolating incidence rates to populations and geographical settings not adequately represented in the included studies.

**Supplementary Table 3. GRADE assessment of evidence quality for the incidence of leptospirosis in LMICs.**

| **GRADE Domain** | **Judgment** | **Reason for Downgrade** | **Comments** |
| --- | --- | --- | --- |
| Risk of bias | Serious ↓ | All three cohort studies (Biggs 2013, Cunha 2022, Banukumar 2016) had unclear overall risk of bias. Confounding-related domains D4 and D5 were unclear in Biggs and Cunha and high risk in Banukumar. Other concerns included baseline outcome status and follow-up completeness. | Proposed downgrade of one level. |
| Inconsistency | Serious ↓ | Substantial between-study heterogeneity remained (I² = 83.12%) in the three-study pooled analysis following exclusion of two influential studies. | Proposed downgrade of one level. |
| Indirectness | Serious ↓ | The final incidence estimate is based on studies from Brazil, India, and Tanzania, limiting applicability to the wider LMIC population. | Proposed downgrade of one level for inference to LMICs generally. |
| Imprecision | Serious ↓ | The pooled incidence was 56.86 cases per 1,000 population per year (95% CI: 42.10-71.62), spanning 29.52 cases per 1,000 per year. | Proposed downgrade of one level if this range is considered meaningfully imprecise. |
| Publication bias | Not assessable statistically | Only three studies contributed to the pooled incidence estimate; funnel-plot asymmetry and Egger’s regression test would be unreliable. | No automatic downgrade; qualitative assessment remains necessary. |
| Overall certainty of evidence | Very Low ↓↓↓↓ | Methodological limitations, residual heterogeneity, restricted applicability and potentially important imprecision reduce confidence in the pooled estimate. | The true incidence may differ substantially from the pooled estimate. |

**Certainty of Evidence Assessment for seroprevalence of leptospirosis in LMICs**

Risk of bias was judged to be serious because of methodological concerns related to sampling representativeness, response rates, and analysis coverage, although 12 studies were classified as having low overall risk of bias and eight as having unclear risk.

Inconsistency was judged to be very serious because of the extremely high heterogeneity, which persisted following subgroup and leave-one-out sensitivity analyses.

Imprecision was considered serious because the 95% confidence interval spanned 15.44 percentage points, indicating potentially meaningful uncertainty in the estimated magnitude of seroprevalence.

Indirectness was not considered serious because the pooled estimate was intended to summarise seroprevalence across the heterogeneous study populations included in the review rather than represent the general population of all LMICs.

Publication bias was judged as undetected, as Egger's regression test did not identify statistically significant small-study effects (p = 0.911), although publication bias could not be definitively excluded.

Overall, the proposed very low certainty rating indicates limited confidence in the pooled seroprevalence estimate, which should be interpreted cautiously given the substantial unexplained heterogeneity and methodological limitations of the included studies.

**Supplementary Table 4. GRADE assessment of evidence quality for the seroprevalence of leptospirosis in LMICs.**

| **GRADE Domain** | **Judgment** | **Reason for Downgrade** | **Comments** |
| --- | --- | --- | --- |
| Risk of bias | Serious ↓ | Of the 20 studies, 12 were assessed as low overall risk of bias and eight as unclear. Concerns included sampling representativeness, response rates, and analysis coverage. | Proposed downgrade of one level. |
| Inconsistency | Very serious ↓↓ | Extremely high heterogeneity (I² = 99.52%) persisted after subgroup and leave-one-out sensitivity analyses. Variation in populations, diagnostic methods, and settings may contribute. | Proposed downgrade of two levels. |
| Indirectness | Not serious | Included community-based, facility-based, and high-risk groups match the descriptive synthesis across included LMIC study populations. | No downgrade; limited generalisability to all LMIC populations. |
| Imprecision | Serious ↓ | Pooled seroprevalence 24.36% (95% CI 16.64-32.08%) spans 15.44 percentage points, judged potentially meaningful for burden interpretation. | Proposed downgrade of one level; review-specific criterion. |
| Publication bias | Undetected | Egger’s test was not significant (p = 0.911). Some visual funnel-plot asymmetry was observed, but evidence was insufficient to strongly suspect publication bias. | No downgrade proposed; publication bias cannot be excluded. |
| Overall certainty of evidence | Very Low ↓↓↓↓ | Methodological limitations, substantial unexplained heterogeneity, and imprecision reduce confidence in the pooled estimate. | The true seroprevalence may differ substantially from the pooled estimate. |

**References:**

1.	Higgins J, Thomas J, Chandler J, Cumpston M, Li T, Page M, et al. Cochrane Handbook for Systematic Reviews of Interventions | Cochrane [Internet]. [cited 2026 Feb 11]. Available from: https://www.cochrane.org/authors/handbooks-and-manuals/handbook
