# Supplementary material for: Unmasking the burden and risk factors of leptospirosis in low- and middle-income countries: a systematic review and meta-analysis
Source: One Health. 2026 Oct 24;23:101632. doi: 10.1016/j.onehlt.2026.101632 (PMC13742084; doi:10.1016/j.onehlt.2026.101632)
Supplement: Supplementary file 4 [file mmc5.docx]

**Supplementary Table 5: Domain-wise summary of risk-factor evidence**

| **Risk-factor domain** | **Number of supporting studies** | **Direction of association** | **Consistency of evidence** |
| --- | --- | --- | --- |
| **Environmental** | 7 | Predominantly positive | **High:** Consistent positive association across studies, although some associations were marginal |
| **Occupational** | 12 | Positive | **High:** Consistently positive associations across occupational exposures, with a few marginal estimates |
| **Animal-related** | 15 | Predominantly positive | **Moderate:** Most studies reported positive associations, but some findings were marginal or mixed |
| **Behavioral** | 7 | Predominantly positive | **Moderate-to-High:** Generally positive associations, with some mixed/marginal findings |
| **Socio-demographic** | 4 | Predominantly positive | **Moderate:** Positive associations were reported, but some findings were mixed |
| **Climate** | 1 | Not formally assessed | **Insufficient:** Descriptive evidence only; no comparative association estimate reported |

**Supplementary Table 6: Structured Risk Factor Evidence Table with analytical outcome**

|  | **Studies with analytical outcome** | | | | | | | |
| --- | --- | --- | --- | --- | --- | --- | --- | --- |
| **Risk Factor Domain** | **Exposure Group** | **Specific Risk Factor** | **Study** | **Setting** | **Population** | **Outcome** | | **Direction** |
|  |  |  |  |  |  | **Reported 95% CI** | **Estimated Type** |  |
| **Environmental** | Flooding/Water Exposure | Flooding / stagnant water | Kawaguchi et al. 2019 | Community | Village populations | OR 2.12 (1.25-3.58) | Adjusted | Positive |
|  |  | Flood history | Suryani et al. 2019 | Community | Hospitalized patients | OR 2.69 (1.23-5.90) | Unadjusted | Positive |
|  |  | Reported household flooding | Gracie et al. 2014 | Community | Neighborhood of Rio de Janeiro | OR 1.10 (0.82-1.46) | Unadjusted | Positive (Marginally) |
|  | Sanitation/Waste | Open sewers / sanitation | Sharvanan et al.2023 | Community | Suspected cases | OR 4.9 (1.2‑19.1) | Adjusted | Positive |
|  |  | Garbage / solid waste | Dias et al.2007 | Community | Residents across drainage basins | OR 1.31 (0.87-1.56) | Adjusted | Positive (marginally) |
|  | Water Quality | Unboiled water | Dung et al. 2022 | Hospital | Clinically suspected patients | OR 2.08 (1.42-3.05) | Adjusted | Positive |
|  |  | Mud puddle / | Suryani et al. 2016 | Community | Hospitalized patients | OR 3.667; 1.238 -10.863 | Unadjusted | Positive |
|  |  | Water ditch | Suryani et al. 2016 | Community | Hospitalized patients | OR 3.0 1.385 -6.499 | Unadjusted | Positive |
| **Occupational** | Agriculture | Farming occupation | Sharvanan . et al. 2023 | Community | Suspected cases | OR 2.7 (0.9‑8.3) | Adjusted | Positive (marginally) |
|  |  | Paddy work | Schønning et al. 2019 | Hospital | Outbreak patients | OR 2.58 (0.991-6.718) | Unadjusted | Positive (marginally) |
|  |  | Paddy field near home | Schønning et al. 2019 | Hospital | Outbreak patients | OR 4.35 (1.61-11.77) | Unadjusted | Positive |
|  |  | Agricultural worker | Shanmughapriya et al. 2014 | Hospital | Suspected cases | OR=1.82 (CI:1.17-2.84) | Adjusted | Positive |
|  |  | Occupational Exposure | de Oliveira et al 2024 | Community | Rural people | OR=1.68 (CI:1.03-3.88), | Adjusted | Positive |
|  |  | Contact with stagnant paddy water (with skin injury | Sahneh et al 2019 | Community | patients diagnosed with leptospirosis | OR=11.21, 95% CI: 3.02, 43.06) | Adjusted | Positive |
|  |  | Recent agricultural work | Vijayachari et al.2004 | Community | School children | OR 1.42 | Unadjusted | Positive |
|  |  | horticulture | Obaidat et al. 2021 | Hospital | Patients were often accompanied by family members, who were invited to participate | OR 1.99(0.89-4.42) | Adjusted | Positive () |
|  |  | Harvesting / | Sugunan et al. 2009 | Community | CHC Rangat patients | OR 5.43 (1.89, 12.93) | Unadjusted | Positive |
|  |  | staying in water | Sugunan et al. 2009 | Community | CHC Rangat patients | OR 3.23 (1.47, 7.21) | Unadjusted | Positive |
|  |  | cleaning sewage | Sugunan et al. 2009 | Community | CHC Rangat patients | OR 3.20 (1.47, 7.21) | Unadjusted | Positive |
|  |  | Outdoor activities related to field work | Kamath et al. 2014 | Community | Confirmed cases | OR 3.95 (1.19-13.0) | Adjusted | Positive |
|  | Slaughterhouse / Abattoir | Slaughterhouse workers | Ntabanganyimana et al 2021 | Community | Adult population | OR 2.40(1.06-5.46) | Adjusted | Positive |
|  | Slaughter house cleaner | Slaughter house cleaner | Anson et al. 2016 | Community | Slaughterhouse worker | OR 5.1(1.8-15.0) | Adjusted | Positive |
| **Animal-related** | Rodent / Small mammal exposure | Rodent exposure | Michael J. et al. 2016 | Community | Livestock keepers | OR 1.8 (1.5-2.0) | Unadjusted | Positive |
|  |  | exposed to rodents | Sohaila 2018 | Community | Suspected cases | OR 10.08( 5.54-18.94) | Unadjusted | Positive |
|  |  | presence of rats | Dias et al 2007 | Community | residents across ten sewage drainage basins | OR=0.93 (C0.63-1.36) | Adjusted | Positive (marginally) |
|  |  | Rat Infestation | Shanmughapriya et al. 2014 | Hospital | Suspected cases | OR 2.60(1.66-4.07) | Unadjusted | Positive |
|  | Livestock / Domestic animals | Pig ownership / livestock | Dung et al.2022 | Hospital | Clinically suspected patients | OR 10.48 (5.05-21.73) | Adjusted | Positive |
|  |  | Cattle in the house | Shanmughapriya et al. 2014 | Hospital | Suspected cases | OR=12.54 (7.39-21.27), | Unadjusted | Positive |
|  |  | Cattle at home of subject | Schoonman et al 2009 | Community | Adult population | OR 1.82(0.92–3.61) | Unadjusted |  |
|  |  | Subject milks cows | Schoonman et al 2009 | Community | Adult population | OR 3.44(1.76–6.75) | Unadjusted |  |
|  |  | animal ownership | Palma et al. 2022 | Community | Low-income urban community, Salvador | OR 0.71 (0.60–0.84) | Adjusted | Positive / Negative |
|  |  | Livestock owner | Hegazy et al. 2020 | Community | Human patients | OR=8.0 (1.55-41.23) | Unadjusted | Positive |
|  |  | Dog in household | Shrestha et al. 2018 | Hospital | Febrile adults | OR 1.1 (0.17-4.) | Unadjusted | Positive |
|  |  | Livestock farmer | Ntabanganyimana et al 2021 | Community | Adult population | OR 4.02 (1.63-9.88) | Adjusted | Positive |
|  |  | animal urine | Shrestha et al. 2018 | Hospital | Febrile adults | OR 2.91 (0.84-9.18) | Unadjusted | Positive |
|  |  | Herder/Farmer | Dreyfus et al 2021 | Community | healthy Bhutanese study participants | OR 1.18 (0.13-10.78) | Adjusted | Positive |
|  |  | Cattle urine exposure / cleaning | Goldwasser et al. 2018 | Hospital | Livestock owners | OR 1.9 (0.91-4.0) | Adjusted | Positive(marginally) |
|  |  | Contact With monkey | Dreyfus et al 2016 | Hospital | All non pregnanant patient and care giver | OR 1.92 (1.18-3.13) | Adjusted | Positive |
|  |  | Domestic animals | de Oliveira et al 2024 | Community | Rural population | OR 2.00 (1.03-3.88). | Adjusted | Positive |
|  |  | Skinning | Dreyfus et al 2021 | Hospital | All non pregnanant patient and care giver | OR 1.92 (1.18-3.13) | Adjusted | Positive |
| **Behavioral** | Personal / Protective | Skin cuts / wounds | Kamath et al. 2014 | Community | Confirmed cases | OR 6.6(2.75-15.86) | Adjusted | Positive |
|  |  | history of cuts or wounds | Sohaila et al. 2018 | Community | Suspected animals | OR 3.26 (2.08-5.09) | Unadjusted | Positive |
|  |  | wounds or cuts on either hands or legs | Awosanya et al 2013 | Community | Febrile and kennel worker | OR 12.0, (1.7 - 14.5) | Unadjusted | Positive |
|  |  | piped water access | Dias et al 2007 | Community | residents across ten sewage drainage basins | OR 1.0 (0.76-1.33) | Adjusted | Positive (marginally) |
|  |  | contact with kennel sewage | Awosanya et al 2013 | Community | Febrile and kennel worker | OR 15.0, 95% CI : 1.5 - 759) | Unadjusted | Positive |
|  |  | face washing in paddy water | Sahneh et al 2019 | Community | patients diagnosed with leptospirosis | OR 11.33  ( 5.12-25.01 | Adjusted | Positive |
|  |  | household trash | Palma et al. 2022 | Community | Low-income urban community, Salvador | OR 1.23 (1.05-1.45) | Adjusted | Positive / Negative |
|  |  | solid garbage nearby | Dias et al 2007 | Community | residents across ten sewage drainage basins | OR 13.1 (0.87-1.56) | Adjusted | Positive(marginally) |
|  |  | Barefoot / soil / water contact | Sugunan et al. 2009 | Community | Febrile patients | OR (2.7 1.1, 6.9) | Adjusted | Positive |
|  | Activity / Recreation | Outdoor activities | Kamath et al. 2014 | Community | Confirmed cases | OR 3.95 (1.19-13.0) | Adjusted | Positive |
|  |  | Pond bathing | Vijayachari et al. 2004 | Community | School children | OR 1.8 | Unadjusted | Positive |
|  |  | Individuals drinking spring or rainwater | Obaidat et al. 2021 | Hospital | Patients were often accompanied by family members, who were invited to participate; | OR 5.69(2.57-12.60 | Adjusted | Positive |
| **Socio-demographic** | Residence / Population | Rural residence | Sohaila et al. 2018 | Community | Urban & rural Pakistan population | OR 4.32 (2.72-6.84) | Unadjusted | Positive |
|  |  | Dwelling near water bodies | Shanmughapriya et al. 2014 | Hospital | Suspected cases | OR=2.9951 (1.89-4.720 | Unadjusted | Positive |
|  |  | low-income slums | Khalil et al. 2021 | Community | Slum communities | OR 1.04 (1.02–1.05) | Unadjusted | Mixed |
|  |  | Black race / | Palma et al. 2022 | Community | Low-income urban community, Salvador | OR 1.72(1.35-2.18) | Adjusted | Positive / Negative |
| **Studies with descriptive outcome** | | | | | | | | |
| **Risk Factor Domain** | **Exposure Group** | **Specific Risk Factor** | **Study** | **Setting** | **Population** | **Estimates Type** | | |
| Animal Related | Livestock/Domestic animals | Dairy cattle contact | Belmaker 2004 | Community | Dairy worker | Descriptive | | |
| Behavioral | Activity / Recreation | Sleeping outside / firewood collection / undercooked meat | Akhvlediani et al. 2019 | Community | Military recruits | Descriptive | | |
| Climate | Climate and environmental factor | High rainfall, high relative humidity and low relative temperature | Cunha et al. 2022 | Community | Suspected leptospirosis case | Descriptive | | |
| Environmental | Water Quality | Presence of rivers / rice fields / wells | Setyaningsih et al. 2022 | Community | Boyolali Regency | Descriptive  18.18% in rivers, 6.67% in rice fields, 18.18% in wells | | |
